# Trifluoromethylation of [AuF_3_(SIMes)]: Preparation and Characterization of [Au(CF_3_)_*x*_F_3−*x*_(SIMes)] (*x=*1–3) Complexes

**DOI:** 10.1002/chem.202002940

**Published:** 2020-10-27

**Authors:** Marlon Winter, Niklas Limberg, Mathias A. Ellwanger, Alberto Pérez‐Bitrián, Karsten Sonnenberg, Simon Steinhauer, Sebastian Riedel

**Affiliations:** ^1^ Fachbereich Biologie, Chemie, Pharmazie Institut für Chemie und Biochemie—Anorganische Chemie Fabeckstr. 34/36 14195 Berlin Germany; ^2^ On leave from: Instituto de Síntesis Química y Catálisis Homogénea (iSQCH) CSIC-Universidad de Zaragoza C/ Pedro Cerbuna 12 50009 Zaragoza Spain

**Keywords:** fluorides, *N*-heterocyclic carbenes, organo gold chemistry, solvent effects, trifluoromethyl ligand

## Abstract

Trifluoromethylation of [AuF_3_(SIMes)] with the Ruppert–Prakash reagent TMSCF_3_ in the presence of CsF yields the product series [Au(CF_3_)_*x*_F_3−*x*_(SIMes)] (*x=*1–3). The degree of trifluoromethylation is solvent dependent and the ratio of the species can be controlled by varying the stoichiometry of the reaction, as evidenced from the ^19^F NMR spectra of the corresponding reaction mixtures. The molecular structures in the solid state of *trans*‐[Au(CF_3_)F_2_(SIMes)] and [Au(CF_3_)_3_(SIMes)] are presented, together with a selective route for the synthesis of the latter complex. Correlation of the calculated SIMes affinity with the carbene carbon chemical shift in the ^13^C NMR spectrum reveals that *trans*‐[Au(CF_3_)F_2_(SIMes)] and [Au(CF_3_)_3_(SIMes)] nicely follow the trend in Lewis acidities of related organo gold(III) complexes. Furthermore, a new correlation between the Au−C_carbene_ bond length of the molecular structure in the solid state and the chemical shift of the carbene carbon in the ^13^C NMR spectrum is presented.

## Graphical abstract

**A new synthetic route** to fluorido trifluoromethyl gold(III) complexes is presented by the trifluoromethylation of [AuF_3_(SIMes)] with TMSCF_3_. The products have a varying number of fluorido ligands substituted by trifluoromethyl ligands. The degree of trifluoromethylation can be controlled by variation of the solvent and the amount of TMSCF_3_.

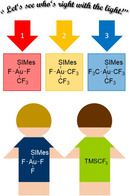

## Introduction

Fluorido organo gold complexes are highly reactive species which take part in a large variety of gold‐catalyzed or ‐mediated reactions.[^1^, ^2^] Their reactivity derives from the relatively low Au−F bond dissociation energy compared to many other known E−F (E=element) bonds.^[3]^ Due to the lack of synthetic routes, their study and application in catalysis was scarce until recent years,[^1^, ^2^] when it was shown that the corresponding fluorido organo gold species can be synthesized in situ. Typical synthetic routes are the oxidation of organo gold(I) complexes with XeF_2_ ^[4]^ or Selectfluor^®^,^[5]^ but fluorination of organo gold(I) complexes with Et_3_N⋅3 HF^[6]^ has also been used. Despite their utility in organometallic reactions, the high reactivity of fluorido gold complexes limits their isolation and characterization.[^2^, ^7^] However, isolation of some derivatives has been reported, either from more accessible halido gold complexes through X/F exchange reactions (X=Cl, Br, I)[^8^, ^9^, ^10^] or by oxidation with XeF_2_.[^11^, ^12^]

*N*‐Heterocyclic carbene (NHC) ligands have attracted increasing attention for the stabilization of highly reactive compounds, for example, complexes that contain transition metals in high oxidation states, due to their steric demand and strong *σ*‐donating properties.^[13]^ Recently, our group reported on the stabilization of AuF_3_ using the SIMes (1,3‐bis(2,4,6‐trimethylphenyl)‐4,5‐dihydroimidazol‐2‐ylidene) ligand.^[14]^ The resulting complex [AuF_3_(SIMes)] contains the highly Lewis acidic AuF_3_ unit in monomeric form and is stable in common organic solvents such as dichloromethane. This is contrary to AuF_3_, which is a fluorine‐bridged polymer in the solid state and reacts unselectively and sometimes violently with most organic solvents.^[15]^

The small size and high electronegativity of fluorine leads to significantly changed properties of a compound when fluorine atoms are introduced.[^16^, ^17^, ^18^] The increased lipophilicity and stability of fluorinated organic compounds compared to non‐fluorinated ones make them useful in a wide range of applications in pharmaceutical^[19]^ and agrochemical^[20]^ industries. The smallest perfluorinated organic moiety is the trifluoromethyl group, which exhibits an increased electron withdrawing property based on its high group electronegativity, which is similar to that of chlorine.^[21]^ However, the selective introduction of CF_3_ groups into organic molecules is still challenging.[^17^, ^18^]

**Scheme 1:**
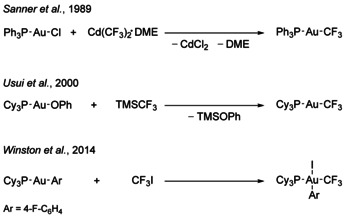
Overview of the different literature‐known pathways for the preparation of trifluoromethyl gold complexes.[^22^, ^23^, ^24^]

**Scheme 2:**
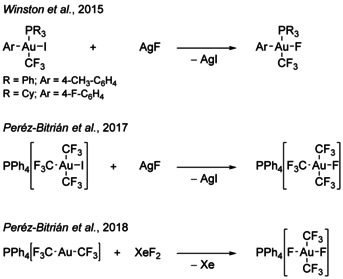
Overview of the preparation of the four literature‐known, isolated fluorido trifluoromethyl gold complexes.[^9^, ^10^, ^12^]

An interesting synthetic approach is the metal‐mediated C−CF_3_ bond formation, a field where gold complexes have played an increasing role since the last decade.[^9^, ^24^, ^25^, ^26^, ^27^] Since the preparation of the first trifluoromethyl gold complex, [Au(CF_3_)Me_2_(PMe_3_)] in 1973,^[28]^ a limited number of such complexes with gold in oxidation states +I, +II and +III have been synthesized.^[29]^ Several synthetic strategies have been established for their preparation (see Scheme 1). They include the oxidative addition of CF_3_I to organo gold(I) complexes,[^10^, ^22^, ^24^, ^28^, ^29^, ^30^, ^31^] and transmetallation reactions of halido organo gold compounds with Cd(CF_3_)_2_⋅DME (DME=1,2‐dimethoxyethane)[^22^, ^31^, ^32^] or of halido or alkoxido organo gold complexes with the Ruppert–Prakash reagent trimethyl(trifluoromethyl)silane (TMSCF_3_) in the presence of a nucleophilic fluoride source when required.[^23^, ^26^, ^33^, ^34^, ^35^, ^36^] Reactions of [Au(CN)_4_]^−^ with ClF in CH_2_Cl_2_ ^[37]^ and [Au(CN)_2_]^−^ with [ClF_4_]^−^ in CHFCl_2_ ^[38]^ were not selective and yielded several products that could not be isolated. Reactions of gold vapors with ^.^CF_3_ radicals^[39]^ or CF_3_X (X=Br, I)^[40]^ were also reported, but are of little synthetic use. The increasing interest in trifluoromethyl gold complexes led to studies on their usage in gold‐mediated C−E (E=C, N, Hal.) bond formations and on further functionalizations of these complexes over the last decade.[^9^, ^10^, ^12^, ^24^, ^25^, ^26^, ^27^, ^29^, ^33^, ^34^, ^35^, ^36^, ^41^, ^42^] Despite the variety of known trifluoromethyl gold complexes, only four complexes containing trifluoromethyl and fluorido ligands at the same gold center have been isolated, namely [Au(CF_3_)(4‐CH_3_−C_6_H_4_)F(PPh_3_)],^[9]^ [Au(CF_3_)(4‐F−C_6_H_4_)F(PCy_3_)],^[9]^ [PPh_4_]‐[Au(CF_3_)_3_F]^[10]^ and [PPh_4_][*trans*‐Au(CF_3_)_2_F_2_]^[12]^ (see Scheme 2). In addition, the series of [Au(CF_3_)_*x*_F_4−*x*_]^−^ anions (*x=*1–3) and other chlorido fluorido trifluoromethyl gold anions,^[37]^ as well as the [*cis*‐Au(CF_3_)(CN)F_2_]^−^ anion^[38]^ were detected by ^19^F NMR spectroscopy. Those complexes can be of interest for further studies on gold‐mediated or ‐catalyzed coupling reactions.

Herein, we report on the synthesis and characterization of the hitherto unknown series [Au(CF_3_)_*x*_F_3−*x*_(SIMes)] (*x=*1–3) by the trifluoromethylation of [AuF_3_(SIMes)]. [Au(CF_3_)_3_(SIMes)] was isolated and the molecular structures in the solid state of *trans*‐[Au(CF_3_)F_2_(SIMes)] and [Au(CF_3_)_3_(SIMes)] will be discussed. These complexes represent the first fluorido trifluoromethyl gold complexes which are prepared by the trifluoromethylation of fluorido gold complexes and not vice versa (cf. Scheme 2).

## Results and Discussion

**Scheme 3:**
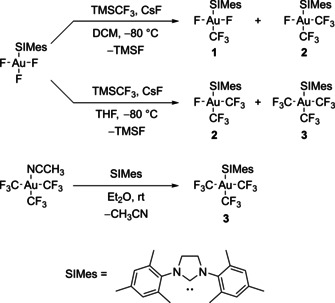
Reaction scheme for the preparation of the target compounds *trans*‐[Au(CF_3_)F_2_(SIMes)] (**1**), *cis*‐[Au(CF_3_)_2_F(SIMes)] (**2**) and [Au(CF_3_)_3_(SIMes)] (**3**). They can be prepared by trifluoromethylation of [AuF_3_(SIMes)] in DCM (top) and THF (middle), and compound **3** can also be prepared by ligand substitution of [Au(CF_3_)_3_(NCCH_3_)] (bottom). In case of the trifluoromethylation, the outcome of the reaction depends on the solvent. In either case, the product ratio can be controlled by the stoichiometry of the reactants, see Table 1 and Table 2 for DCM and THF, respectively.

The molecular structure of [AuF_3_(SIMes)] in the solid state reveals that the Au−F bond with the fluorido ligand in *trans* position to the SIMes ligand is about 5 pm longer than those to the *cis*‐fluorido ligands.^[14]^ In accordance with this finding, a selective substitution of the *trans*‐fluorido ligand by a chlorido or a pentafluoridoorthotellurato (OTeF_5_) ligand was recently reported by us.^[43]^ Hence, the trifluoromethylation of [AuF_3_(SIMes)] is expected to yield *trans*‐[Au(CF_3_)F_2_(SIMes)] (**1**). Indeed, when TMSCF_3_ is condensed into a DCM solution of [AuF_3_(SIMes)] at −80 °C in the presence of the nucleophilic fluoride source CsF, compound **1** is formed. ^19^F NMR spectroscopy investigations show that first only compound **1** is formed, but during the consumption of [AuF_3_(SIMes)], a second substitution reaction of a fluorido ligand by a trifluoromethyl group is observed, forming *cis*‐[Au(CF_3_)_2_F(SIMes)] (**2**) in solution (cf. Scheme 3). After a few hours, no [AuF_3_(SIMes)] is left and the ratio of the products stays constant for several days, even though unreacted TMSCF_3_ is left in the reaction mixture (see Supporting Information, Figures S9–S15). Therefore, the reaction proceeds rather fast and the reaction time does not have a relevant influence on the product ratio. Instead, the stoichiometry of the reactants has a decisive impact on the ratio between **1** and **2**. As expected, the formation of *trans*‐[Au(CF_3_)F_2_(SIMes)] (**1**) is favored by ≈0.5 equivalents of TMSCF_3_, while an excess of TMSCF_3_ increases the amount of *cis*‐[Au(CF_3_)_2_F(SIMes)] (**2**), as shown in Table 1. HCF_3_ and *trans*‐[AuClF_2_(SIMes)] are formed as by‐products, the presence of the former being probably due to side reactions of the highly basic transient CF_3_^−^ anion with any proton sources, for example, from the solvent,[^44^, ^45^, ^46^] while the latter is formed by a chlorine/fluorine exchange reaction between [AuF_3_(SIMes)] and DCM, possibly promoted by the fluoride anions of CsF.^[14]^ The amount of by‐products depends on the stoichiometry. The more TMSCF_3_ is used, the more HCF_3_ is formed, while less [AuClF_2_(SIMes)] is present (see Supporting Information, Figures S10, S13 and S15). Compounds **1** and **2** partially decompose in solution at room temperature within a few days, leading to the formation of elemental gold. However, the ^19^F NMR spectra still show signals of the products after several weeks.

**Table 1:** Product ratio dependence of the stoichiometric factors in the reaction between [AuF_3_(SIMes)] and TMSCF_3_ in DCM determined by the integral ratios of the signals in the ^19^F NMR spectra (see Supporting Information, Figures S9–S15). Note that the amount of TMSCF_3_ can slightly deviate from the values listed in the table, due to the inherent uncertainty of the used manometer for determining the pressure of TMSCF_3_.

eq. ([AuF_3_(SIMes)])	eq. (TMSCF_3_)	*trans*‐[Au(CF_3_) : *cis*‐[Au(CF_3_)_2_ F_2_(SIMes)] (**1**) : F(SIMes)] (**2**)
	
1	0.5	7 : 1
1	1	2 : 1
1	5	1 : 1

If the trifluoromethylation of [AuF_3_(SIMes)] is performed in THF, compound **1** is not observed in the ^19^F NMR spectrum of the reaction mixture. Instead, compound **2** and the three times substituted complex [Au(CF_3_)_3_(SIMes)] (**3**) are formed (cf. Scheme 3). The reason for the formation of higher substituted products in THF is most likely the better solubility of CsF in THF compared to DCM. This leads to an enhanced activation of the TMSCF_3_ forming a pentacoordinated silicon(IV) anion, which acts as a highly potent CF_3_ transfer reagent, as shown by Naumann et al.^[46]^ In contrast to the reaction in DCM, no TMSCF_3_ is left in the reaction mixture after a few hours (see Supporting Information, Figures S16 and S17). The stoichiometry of the reactants has a significant influence on the product ratio. When [AuF_3_(SIMes)] and TMSCF_3_ are used in roughly a 1:1 ratio, compounds **2** and **3** are formed in almost equal amounts. If only about half an equivalent of TMSCF_3_ is used, five times more **2** than **3** is formed (cf. Table 2). The transfer of two or three CF_3_ groups, even though not more than one equivalent of TMSCF_3_ is used, can most likely be explained by the lower solubility of [AuF_3_(SIMes)] in THF. Furthermore, the formation of HCF_3_ and the [Au(CF_3_)_4_]^−^ anion as by‐products is observed, which increases with the amounts of TMSCF_3_ (see Supporting Information, Figures S16 and S17). The existence of the former is probably due to a reaction of the highly basic CF_3_^−^ anion, which is abstracted from the pentacoordinated silicon(IV) anion, with proton sources in the reaction mixture,[^44^, ^46^] while the latter is possibly formed by the trifluoromethylation of traces of the [AuF_4_]^−^ anion.

**Table 2:** Product ratio dependence of the stoichiometric factors in the reaction between [AuF_3_(SIMes)] and TMSCF_3_ in THF determined by the integral ratios of the signals in the ^19^F NMR spectra (see Supporting Information, Figures S16 and S17). Note that the amount of TMSCF_3_ can slightly deviate from the values listed in the table, due to the inherent uncertainty of the used manometer for determining the pressure of TMSCF_3_.

eq. ([AuF_3_(SIMes)])	eq. (TMSCF_3_)	*cis*‐[Au(CF_3_)_2_ : [Au(CF_3_)_3_ F(SIMes)] (**2**) : (SIMes)] (**3**)
	
1	0.5	5 : 1
1	1	1 : 1

[Au(CF_3_)_3_(SIMes)] can be isolated via a different route, starting from the literature‐known complex [Au(CF_3_)_3_(NCCH_3_)]^[42]^ by substitution of the acetonitrile ligand with SIMes. A room temperature solution of **3** in DCM or THF is stable for several weeks. In pure form, compound **3** can be stored under an argon atmosphere at room temperature for months and it is stable under air for several days without decomposition. The synthetic routes for the preparation of the target compounds *trans*‐[Au(CF_3_)F_2_(SIMes)] (**1**), *cis*‐[Au(CF_3_)_2_F(SIMes)] (**2**) and [Au(CF_3_)_3_(SIMes)] (**3**) are summarized in Scheme 3 and an overview on the chemical shifts in the ^19^F NMR spectra, which will be discussed below, is given in Table 3.

**Table 3:** ^19^F NMR spectroscopic data of the target compounds *trans*‐[Au(CF_3_)F_2_(SIMes) (**1**), *cis*‐[Au(CF_3_)_2_F(SIMes)] (**2**) and [Au(CF_3_)_3_(SIMes)] (**3**). The subscripts c and t stand for CF_3_ groups in *cis* or *trans* position to the SIMes ligand, respectively.^[a]^

Compound	*δ*(CF_3,c_)	*δ*(CF_3,t_)	*δ*(F)	^3^ *J*(^19^F,^19^F), *cis*	^3^ *J*(^19^F,^19^F), *trans*	^4^ *J*(^19^F,^19^F)
*trans*‐[Au(CF_3_)F_2_(SIMes)] (**1**)		−46.5	−329.7	18		
*cis*‐[Au(CF_3_)_2_F(SIMes)] (**2**)	−23.9	−41.2	−254.0	14	57	7
[Au(CF_3_)_3_(SIMes)] (**3**)	−31.5	−34.3				7

[a] Chemical shifts are given in ppm and coupling constants in Hz.

**Figure 1:**
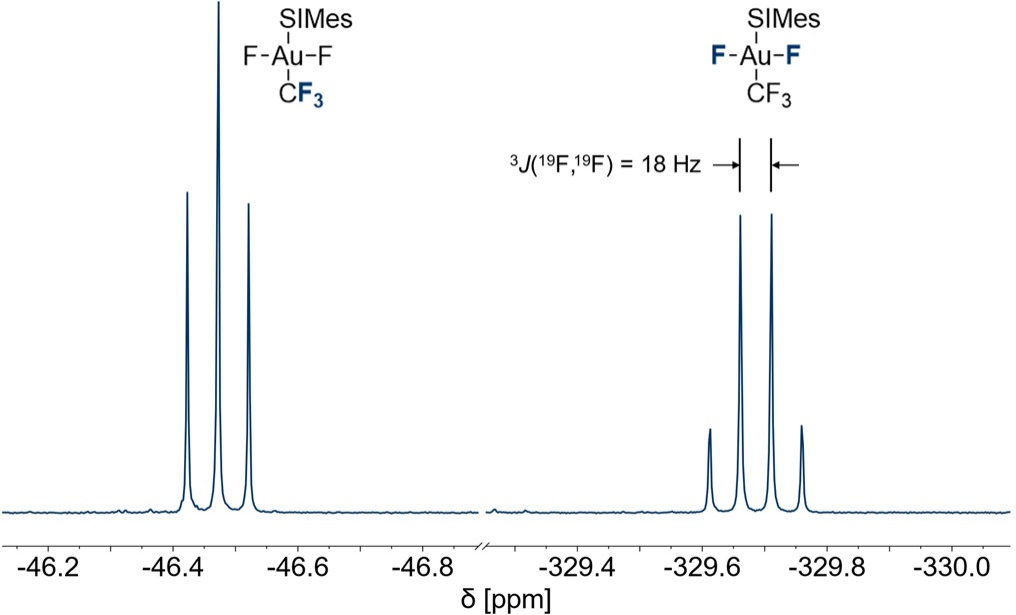
^19^F NMR spectrum (377 MHz, CD_2_Cl_2_, 20 °C) of *trans*‐[Au(CF_3_)F_2_(SIMes)] (**1**).

The ^19^F NMR spectrum of *trans*‐[Au(CF_3_)F_2_(SIMes)] (**1**) shows a triplet at −46.5 ppm and a quartet at −329.7 ppm with a ^3^ *J*(^19^F,^19^F) coupling constant of 18 Hz, which correspond to the trifluoromethyl and the two fluorido ligands, respectively (see Figure 1). The former resonance is in the upfield range of chemical shifts of the corresponding trifluoromethyl groups in literature‐known fluorido trifluoromethyl gold(III) complexes[^9^, ^10^, ^12^, ^37^] and almost identical to the one in the [*trans*‐Au(CF_3_)_2_F_2_]^−^ anion (*δ*=−46.2 ppm).^[12]^ The latter is 4 ppm −19 ppm upfield shifted compared to the *cis*‐fluorido ligands in the literature‐known *trans*‐[AuF_2_X(SIMes)] (X=Cl, F, OTeF_5_) complexes.[^14^, ^43^] An interesting feature of metal complexes with NHC ligands is the chemical shift of the carbene carbon atom in the ^13^C NMR spectra, which was proven to be a measure of the Lewis acidity of the metal center.[^14^, ^43^, ^47^] In the ^1^H,^13^C HMBC NMR spectrum of **1**, the resonance of the carbene carbon atom was detected at 192.3 ppm, which is 26 ppm −45 ppm downfield shifted compared to the literature‐known *trans*‐[AuF_2_X(SIMes)] (X=Cl, F, OTeF_5_)[^14^, ^43^] complexes (cf. Table 4) due to the weaker Lewis acidity of the gold center in compound **1**. This is in good agreement with the corresponding Au−C_carbene_ bond lengths in the solid state and the calculated dissociation energy of the Au−C_carbene_ bond, as discussed in detail below.

**Table 4:** Comparison of the calculated SIMes affinities (−Δ_*r*_ *G*_diss_), gold carbon distances in the molecular structures in the solid state (*r*(Au−C_carbene_)) and chemical shifts of the carbene carbon atoms in the ^13^C NMR spectra (*δ*(^13^C_carbene_) of *trans*‐[Au(CF_3_)F_2_(SIMes)] (**1**) and similar, literature‐known compounds.

Compound	−Δ_*r*_ *G*_diss_ [kJ mol^−1^]	*r*(Au−C_carbene_) [pm]	*δ*(^13^C_carbene_) [ppm]
*trans*‐[Au(CF_3_)F_2_(SIMes)] (**1**)	251	203.5(9)	192
*trans*‐[AuClF_2_(SIMes)]	342	200.8(3)	166
[AuF_3_(SIMes)]	405	197.3(1)	152
*trans*‐[AuF_2_(OTeF_5_)(SIMes)]	432	196.9(5)	149

**Figure 2:**
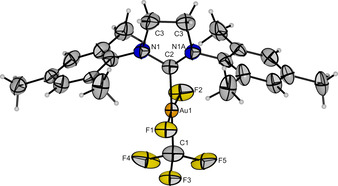
Molecular structure of *trans*‐[Au(CF_3_)F_2_(SIMes)] in the solid state. Disorders of the SIMes ligand and the CF_3_ group are omitted for clarity (cf. Supporting Information, Figure S1). Thermal ellipsoids are set at 50 % probability. Bond lengths [pm] to the central gold atom: 193.2(5) (F1−Au1), 193.2(10) (F2−Au1), 203.6(10) (C1−Au1), 203.5(9) (C2−Au1).

Single crystals of compound **1** suitable for X‐ray diffraction were obtained by slow vapor diffusion of *n*‐pentane at 5 °C into a DCM solution of the reaction between [AuF_3_(SIMes)] and TMSCF_3_. Compound **1** crystallizes in the orthorhombic space group *Pnma* with a square planar coordination around the gold center (see Figure 2). The Au−F bond lengths (193.2(5) pm, 193.2(10) pm) are comparable to the corresponding Au−F bond lengths in the starting compound [AuF_3_(SIMes)] (191.6(1) pm, 192.1(1) pm).^[14]^ The Au−CF_3_ bond length (203.6(10) pm) is similar to those in the literature‐known anion *trans*‐[Au(CF_3_)_2_F_2_]^−^ (205.5(5) pm, 205.3(5) pm)^[12]^ and of the corresponding Au−CF_3_ bond in the neutral isocyanide complex [Au(CF_3_)_3_(CNC(CH_3_)_3_)] (205.8(5) pm).^[42]^ The Au−C_carbene_ bond length (203.5(9) pm) is slightly elongated compared to *trans*‐[AuClF_2_(SIMes)] (200.8(3) pm),^[43]^ [AuF_3_(SIMes)] (197.3(1) pm)^[14]^ and *trans*‐[AuF_2_(OTeF_5_)(SIMes)] (196.9(5) pm).^[43]^ This trend can be explained by the strong *trans*‐influence of the CF_3_ group, which is discussed in detail below (cf. Table 4 and Figure 7).

**Figure 3:**
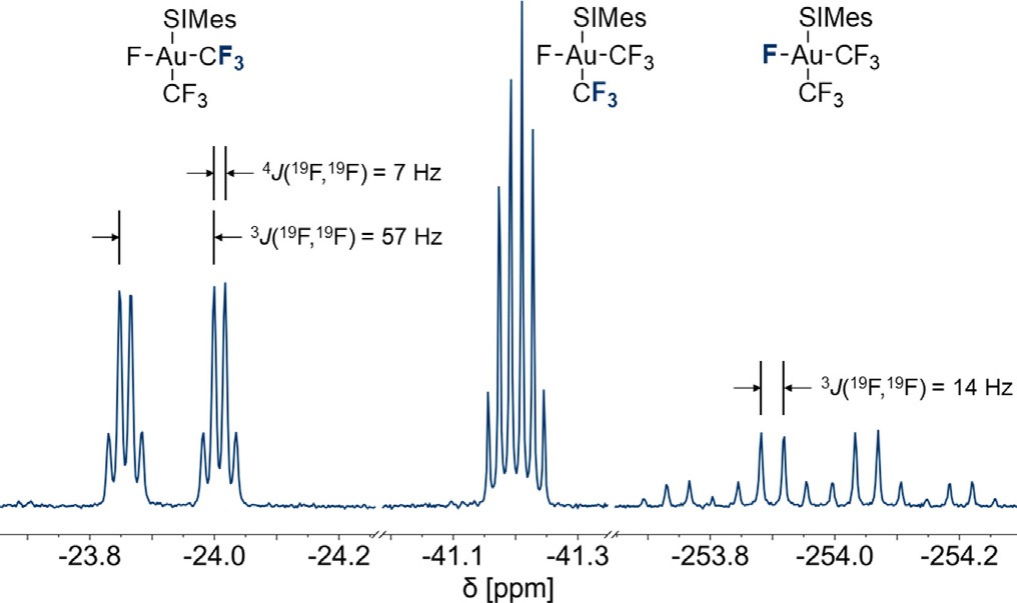
^19^F NMR spectrum (377 MHz, CD_2_Cl_2_, 20 °C) of *cis*‐[Au(CF_3_)_2_F(SIMes)] (**2**).

The ^19^F NMR spectrum of *cis*‐[Au(CF_3_)_2_F(SIMes)] (**2**) (see Figure 3) consists of two doublets of quartets at −23.9 ppm and −41.2 ppm, where the latter appears to be a sextet due to the coupling constants of 7 Hz and 14 Hz, and a quartet of quartets at −254.0 ppm in an integral ratio of 3:3:1. The latter resonance belongs to the remaining fluorido ligand, while the two other signals are due to the two chemically inequivalent CF_3_ groups. The chemical shifts are all similar to the literature‐known [Au(CF_3_)_3_F]^−^ anion.^[10]^ The ^3^ *J*(^19^F,^19^F) coupling constants between the fluorine nuclei of the trifluoromethyl groups and the fluorido ligand are 57 Hz and 14 Hz for the signals at −23.9 ppm and −41.2 ppm, respectively. It is known that *trans* coupling constants are usually larger than *cis* coupling constants,^[37]^ for example, in the [Au(CF_3_)_3_F]^−^ anion, the coupling constants are 55.8 Hz (*trans*) and 12.3 Hz (*cis*).^[10]^ Furthermore, the coupling constant of 14 Hz is in good agreement with the ^3^ *J*(^19^F,^19^F) *cis* coupling in **1** (18 Hz) and the literature‐known *trans*‐[Au(CF_3_)_2_F_2_]^−^ anion (16.5 Hz).^[12]^ Therefore, the signals at −23.9 ppm and −41.2 ppm can be assigned to the CF_3_ groups *cis* and *trans* to the carbene ligand, respectively (cf. Figure 3 and Table 3).

**Figure 4:**
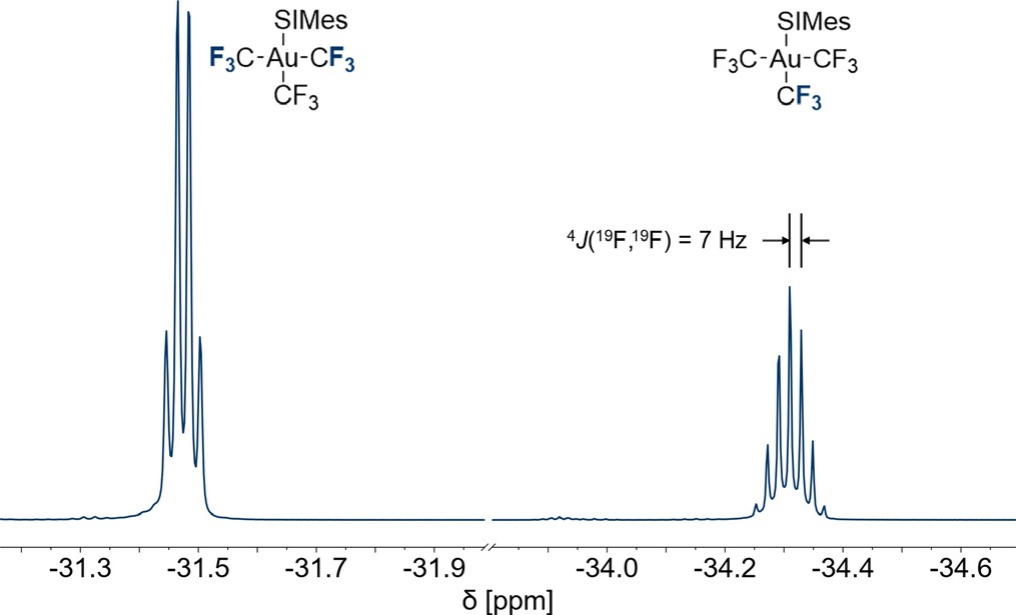
^19^F NMR spectrum (377 MHz, CD_2_Cl_2_, 21 °C) of [Au(CF_3_)_3_(SIMes)] (**3**).

Figure 4 shows the ^19^F NMR spectrum of [Au(CF_3_)_3_(SIMes)] (**3**), which consists of a quartet at −31.5 ppm and a septet at −34.3 ppm with an integral ratio of 2:1. The quartet belongs to the two *trans*‐positioned CF_3_ groups and the septet to the CF_3_ group in *trans*‐position to the SIMes ligand. The coupling constant of 7 Hz is identical to the ^4^ *J*(^19^F,^19^F) coupling constant of the two CF_3_ groups in compound **2** and fits nicely within the range of neutral complexes containing the Au(CF_3_)_3_ fragment prepared by Menjón et al. (6.0–7.5 Hz).^[42]^ Compared to the acetonitrile complex [Au(CF_3_)_3_(NCCH_3_)], which was used as a starting material for the selective synthesis of compound **3**, the relative position of the two resonances are interchanged.^[42]^ In the ^1^H,^13^C HMBC NMR spectrum, the resonance of the carbene carbon atom in compound **3** is observed at 191.4 ppm, which is only 1 ppm upfield shifted compared to compound **1**, and thus points towards a similar Lewis acidity (cf. discussion below).

**Figure 5:**
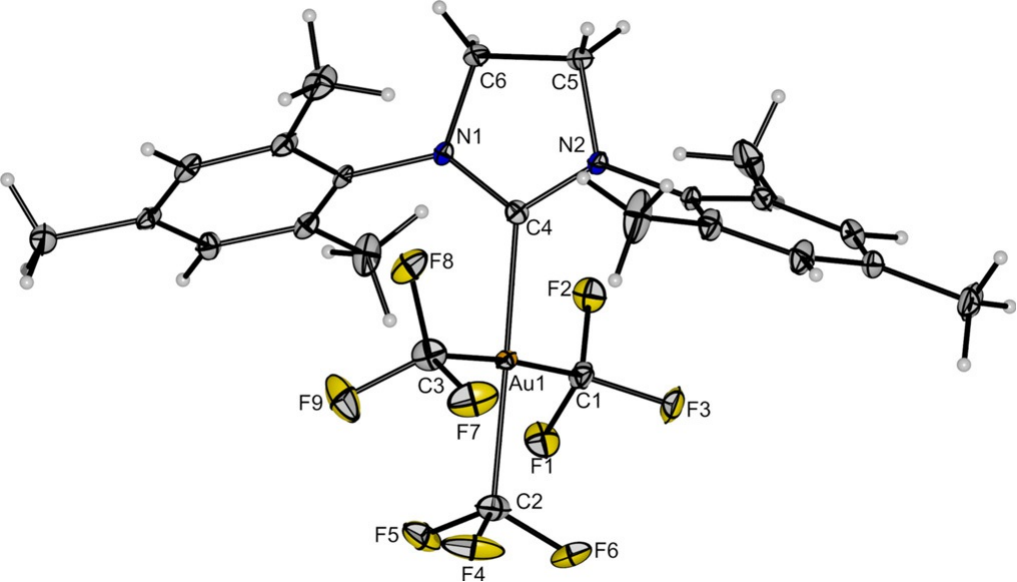
Molecular structure of [Au(CF_3_)_3_(SIMes)]⋅0.5 CH_2_Cl_2_ in the solid state. The disordered CH_2_Cl_2_ molecule is omitted for clarity. Thermal ellipsoids are set at 50 % probability. Bond lengths [pm] to the central gold atom: 208.6(3) (C1−Au1), 207.8(3) (C2−Au1), 208.3(3) (C3−Au1), 208.1(2) (C4−Au1).

In order to obtain single crystals of compound **3** suitable for X‐ray diffraction, a pure sample of **3** prepared by the reaction between [Au(CF_3_)_3_(NCCH_3_)] and SIMes was dissolved in dichloromethane, chloroform, acetone or tetrahydrofuran, and each of the solutions was layered by *n*‐hexane at 5 °C. From all four solvents, compound **3** crystallizes in the monoclinic space group *P*2_1_/*c* with a square planar coordination around the gold center and contains half a co‐crystallized, disordered solvent molecule. Since the four structures are homeotypic (see Supporting Information, Figures S3–S6), the following discussion is based on the structural data of [Au(CF_3_)_3_(SIMes)]⋅0.5 CH_2_Cl_2_ (Figure 5). The Au−CF_3_ bond lengths to the two CF_3_ groups in *cis* position to the SIMes ligand (208.3(3) pm, 208.6(3) pm) are in the typical range for neutral [Au(CF_3_)_3_L] complexes (207.4(9)–209.8(2) pm)^[42]^ and similar to those in [NBu_4_][Au(CF_3_)_4_] (207.5(6) pm, 208.5(7) pm).^[34]^ The Au−CF_3_ bond length of the CF_3_ group *trans* to the SIMes ligand (207.8(3) pm) is comparable to the other two Au−CF_3_ bonds and in the upper range of neutral [Au(CF_3_)_3_L] complexes (200.1(3)–209.0(3) pm).^[42]^ The Au−C bond to the SIMes ligand (208.1(2) pm) is slightly longer than in compound **1** (203.5(9) pm; cf. Figure 2) and 3–11 pm longer than in other literature‐known [AuX_3_(SIMes)] complexes (X=F, Cl, Br).[^14^, ^48^] The elongation of this Au−C bond is due to the strong *trans*‐influence of the CF_3_ group compared to the halides,[^49^, ^50^, ^51^] which also explains why the Au−CF_3_ bond lengths of the two *trans*‐standing CF_3_ groups are usually longer than that of the CF_3_ group *trans* to the donor ligand in similar complexes.^[42]^

**Figure 6:**
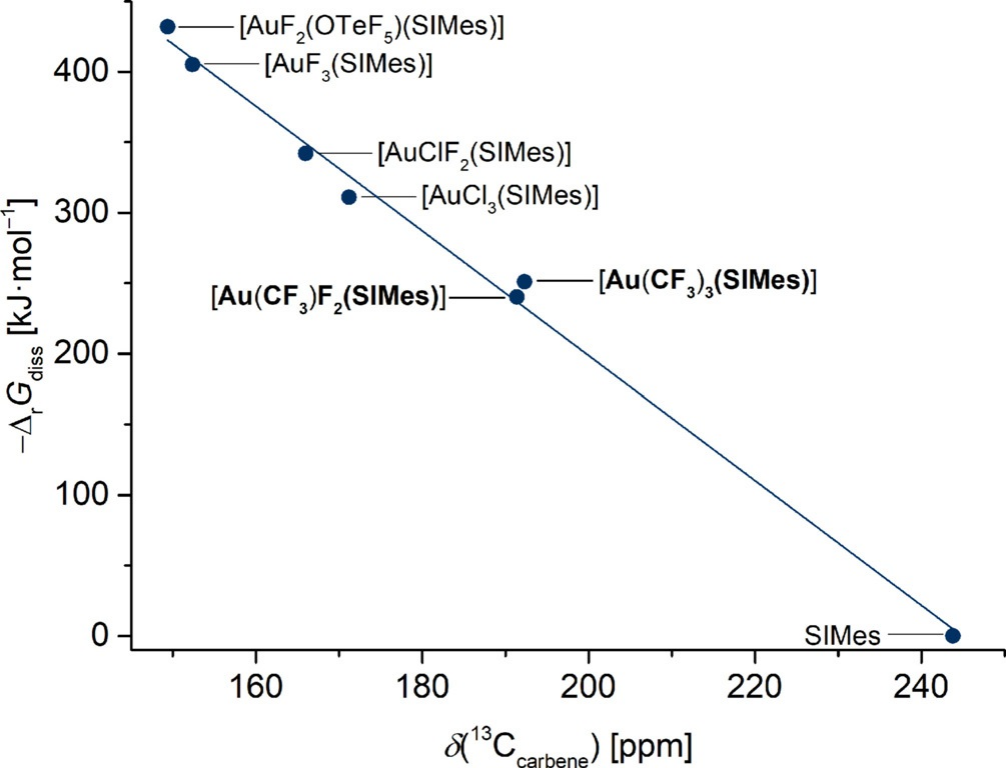
Correlation between the calculated SIMes affinity (−Δ_*r*_ *G*_diss_) with the chemical shifts of the carbene carbon atoms in the ^13^C NMR spectra (*δ*(^13^C_carbene_)) of *trans*‐[Au(CF_3_)F_2_(SIMes)] (**1**), [Au(CF_3_)_3_(SIMes)] (**3**) (both highlighted in bold) and similar, literature‐known compounds.^[43]^ The ^13^C chemical shift of uncoordinated SIMes, which has by definition a SIMes affinity of 0 kJ mol^−1^, is also included.^[55]^

Recently, our group reported on the calculation and experimental access to the “SIMes affinity” of gold(III) moieties. Therein, the Gibbs free energy Δ_r_ *G*_diss_ of the dissociation of the SIMes ligand from [AuF_2_X(SIMes)] and [AuX_3_(SIMes)] complexes (X=Cl, F, OTeF_5_) forming SIMes and the corresponding [AuF_2_X] or [AuX_3_] fragment on the RI‐B3LYP‐D3/def2‐TZVPP level of theory at 0 °C were calculated.^[43]^ This SIMes affinity can be correlated with the chemical shift of the carbene carbon atom in the ^13^C NMR spectrum. We found a nearly linear relationship between an increase of the SIMes affinity and an upfield shift in the ^13^C NMR spectrum.^[43]^ We have now determined the SIMes affinities of *trans*‐[Au(CF_3_)F_2_(SIMes)] (**1**) and [Au(CF_3_)_3_(SIMes)] (**3**) to be 251 kJ mol^−1^ and 240 kJ mol^−1^, respectively. The corresponding complexes with Cl, F and/or OTeF_5_ ligands exhibit significantly higher SIMes affinities, ranging from 300 kJ mol^−1^ to 430 kJ mol^−1^. This trend is in accordance with the chemical shifts of the carbene carbon atom in the ^13^C NMR spectra, as depicted in Figure 6. Theoretical studies show that a downfield shift of the carbene carbon atom in gold complexes results from a deshielding of the carbene carbon atom by ligands with a strong *trans*‐influence.^[52]^ Our results are in good agreement with the literature, where the trifluoromethyl group was determined to have a *trans*‐influence in the order of the methyl group, which is one of the strongest *trans*‐influencing ligands, and a much stronger *trans*‐influence than the halides.[^49^, ^50^, ^51^, ^53^] A recent study on hydrido gold(III) complexes showed that also the *cis*‐influence can change the chemical shift.^[54]^ However, this is not the case in our trifluoromethyl gold complexes, since compounds **1** and **3** have similar SIMes affinities and ^13^C_carbene_ chemical shifts.

**Figure 7:**
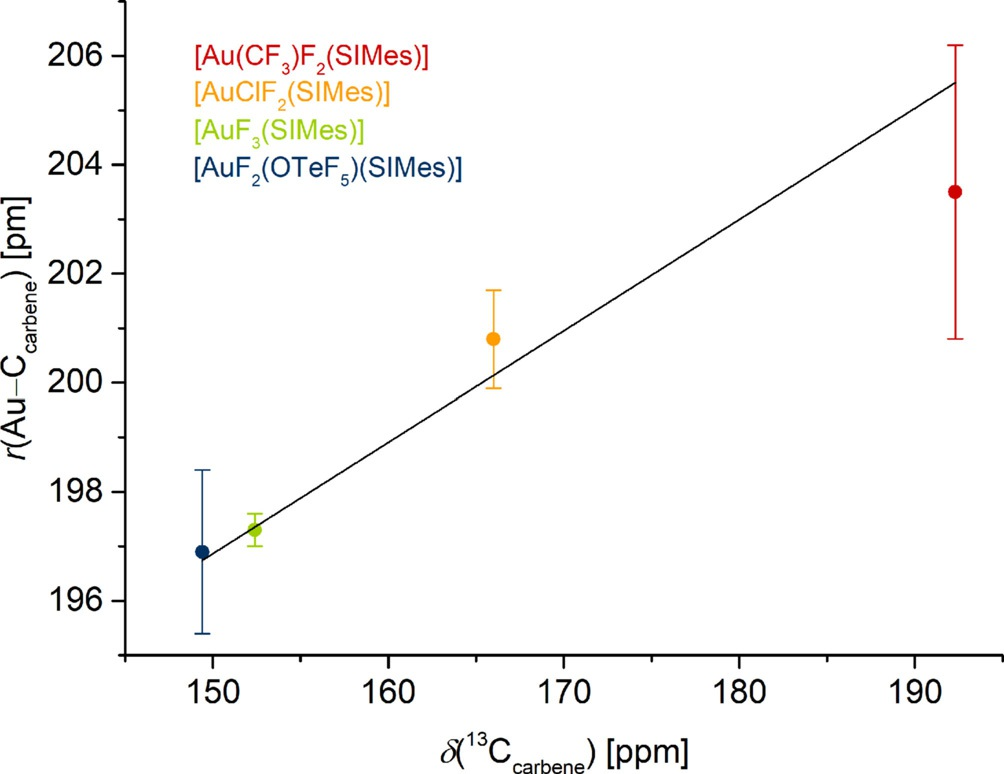
Correlation between the gold carbon distances (*r*(Au−C_carbene_)) in the molecular structures in the solid state with the chemical shifts of the carbene carbon atoms in the ^13^C NMR spectra (*δ*(^13^C_carbene_)) of *trans*‐[Au(CF_3_)F_2_(SIMes)] (**1**) (red) and similar, literature‐known compounds. The error bars of the *y*‐axis equal three times the estimated standard deviation (the error in the last digit written in brackets in Table 4).

Regarding the series of [AuF_2_X(SIMes)] complexes (X=CF_3_, Cl, F, OTeF_5_), the theoretically determined SIMes affinity is a good measure for the strength of the Au−C_carbene_ bond, which is inversely proportional to the experimentally determined Au−C_carbene_ bond length in the molecular structures in the solid state. Table 4 lists the SIMes affinities, gold carbon distances and ^13^C chemical shifts of the carbene carbon atom in these complexes. Figure 7 shows the nearly linear relationship between the Au−C_carbene_ bond length and the chemical shift of the carbene carbon atom, which underlines the use of the calculated Gibbs free energies as a measure of the strength of the Au−C_carbene_ bond. The *trans*‐influence rises in the order OTeF_5_<F<Cl≪CF_3_. In summary, the higher *trans*‐influence of the CF_3_ ligand in **1** leads to a lower Lewis acidity of the gold(III) center, resulting in a longer Au−C_carbene_ bond length and a deshielding of the carbene carbon atom.

## Conclusions

In summary, the trifluoromethylation of [AuF_3_(SIMes)] with TMSCF_3_ to result in unprecedented products of the series [Au(CF_3_)_*x*_F_3−*x*_(SIMes)] (*x=*1–3) in different solvents has been described, being the first synthetic route for those kind of complexes starting from fluorido gold complexes. When the reaction is performed in DCM, *trans*‐[Au(CF_3_)F_2_(SIMes)] (**1**) and *cis*‐[Au(CF_3_)_2_F(SIMes)] (**2**) are formed, while the reaction in THF yields a mixture of *cis*‐[Au(CF_3_)_2_F(SIMes)] (**2**) and [Au(CF_3_)_3_(SIMes)] (**3**). In both cases, the product ratio does not change significantly with longer reaction times, but it can be controlled by the stoichiometry of the reaction, as evidenced by the ^19^F NMR spectra. More TMSCF_3_ leads to a larger amount of the higher‐substituted product. Furthermore, for the selective preparation of compound **3**, an alternative synthetic route starting from [Au(CF_3_)_3_(NCCH_3_)] is described. The ^13^C NMR shifts of the carbene carbon atoms in compounds **1** and **3** can be correlated with the calculated dissociation energies of the Au−C_carbene_ bond, revealing a significantly lower Lewis acidity compared to literature‐known [AuF_2_X(SIMes)] or [AuX_3_(SIMes)] complexes (X=Cl, F, OTeF_5_). These dissociation energies are in accordance with the trend in the Au−C_carbene_ bond lengths in the [AuF_2_X(SIMes)] series. This article offers a new synthetic route to the rare group of fluorido trifluoromethyl gold(III) complexes which could find interesting properties for gold‐mediated coupling reactions.

## Experimental Section

CAUTION! Strong Oxidizers! All reactions should be performed under strictly anhydrous conditions. The combination of AuF_3_ with organic materials can lead to violent reactions. On contact with only small amounts of moisture, all used fluoride‐containing compounds decompose under the formation of HF. Therefore, appropriate treatment procedures should be available in case of a contamination with HF‐containing solutions.

**Materials, chemicals and procedures**: All experiments were performed under rigorous exclusion of air and moisture using standard Schlenk techniques. All solids were handled in an MBRAUN UNIlab plus glovebox with an argon atmosphere (O_2_<0.5 ppm, H_2_O<0.5 ppm). Solvents were dried using freshly ground CaH_2_ in case of CH_2_Cl_2_, CHCl_3_, CD_2_Cl_2_ and *ortho*‐difluorobenzene, SICAPENT® in case of CH_3_CN, potassium in case of Et_2_O and sodium in case of THF, *n*‐pentane and *n*‐hexane. Acetone was distilled prior to use. All solvents were stored over 4 Å molecular sieves, except for CH_3_CN, which was stored over 3 Å molecular sieves. AuF_3_,^[56]^ 1,3‐bis(2,4,6‐trimethylphenyl)‐4,5‐dihydroimidazol‐2‐ylidene (SIMes)^[57]^ and [Au(CF_3_)_3_(NCCH_3_)]^[42]^ were prepared using literature‐known methods. Raman spectra were recorded at room temperature using a Bruker MultiRAM FT‐Raman spectrometer with a 1064 nm wavelength ND:YAG laser. The spectra were measured directly inside the reaction flask with a laser power of 30 mW and 64 scans with a resolution of 2 cm^−1^. IR spectra were measured at room temperature inside a glovebox under argon atmosphere using a Bruker ALPHA FTIR spectrometer with a diamond ATR attachment with 32 scans and a resolution of 4 cm^−1^. Raman and IR spectra were processed using OPUS 7.5 and Origin 9.1^[58]^ was used for their graphical representation. NMR spectra were recorded using a JEOL 400 MHz ECZ or ECS spectrometer and all chemical shifts are referenced as defined in the IUPAC recommendations of 2001.^[59]^ MestReNova 14.0 was used to process the spectra and for their graphical representation. X‐ray diffraction measurements were performed on a Bruker D8 Venture with MoK_*α*_ (*λ*=0.71073 nm) radiation at 100 K. Single crystals were picked in perfluoroether oil at 0 °C under nitrogen atmosphere and mounted on a 0.15 mm Mitegen micromount. They were solved using the ShelXT^[60]^ structure solution program with intrinsic phasing and were refined with the refinement package ShelXL^[61]^ using least squares minimizations by using the program OLEX2.^[62]^ Diamond 3 and POV‐Ray 3.7 were used for their graphical representation. Quantum chemical calculations were performed using the functional B3LYP^[63]^ with RI^[64]^ and Grimme‐D3^[65]^ and the basis set def2‐TZVPP^[66]^ as incorporated in TURBOMOLE.^[67]^

**Preparation of [AuF_3_(SIMes)]**: The synthesis of [AuF_3_(SIMes)] was based on a literature‐known procedure^[43]^ with slight deviations and upscaling of the synthesis. In a typical experiment, AuF_3_ (400 mg, 1.58 mmol, 1 equiv.) and SIMes (483 mg, 1.58 mmol, 1 equiv.) were dissolved in dichloromethane (20 mL) and stirred for 30 minutes at −80 °C. *Ortho*‐difluorobenzene (20 mL) was added and the mixture was stirred for 30 minutes at −80 °C. Volatiles were removed under reduced pressure at −40 °C until a dark grey precipitate was formed. The resulting yellowish solution was filtered off. *ortho*‐Difluorobenzene (20 mL) was added to the solid residue, the mixture was stirred for 30 minutes at −40 °C and the solution was filtered off. This washing process was repeated until the filtrated solution was colorless (usually, three times were sufficient). Dichloromethane (20 mL) was added and the mixture was stirred for 30 minutes at −80 °C, yielding a dark solution. The mixture was filtered through a hydrophobic PTFE filter (0.2 μm). *ortho*‐Difluorobenzene (20 mL) was added to the colorless solution, the mixture was stirred for 30 minutes at −80 °C and volatiles were removed at −40 °C until a colorless precipitate was formed. The colorless solution was filtered off and residual solvent was removed under reduced pressure. The product (206 mg, 0.368 mmol, 23 %) was obtained as a colorless powder. ^1^H NMR (400 MHz, CD_2_Cl_2_, 21 °C): *δ*=7.05 (s, 4 H, *meta*‐CH), 4.28 (s, 4 H, NCH_2_CH_2_N), 2.34 (s, 6 H, *para*‐CH_3_), 2.33 (s, 12 H, *ortho*‐CH_3_) ppm. ^19^F NMR (377 MHz, CD_2_Cl_2_, 21 °C): *δ*=−216.9 (t, 1F, *trans*‐F, ^2^ *J*(^19^F,^19^F)=49 Hz), −315.7 (d, 2F, *cis*‐F) ppm.

**Trifluoromethylation of [AuF_3_(SIMes)] in dichloromethane**: In a typical experiment, [AuF_3_(SIMes)] (10 mg, 17.8 μmol, 1 equiv.) and CsF (2.7 mg, 17.8 μmol, 1 equiv.) were dissolved in dichloromethane (1 mL) and at −196 °C, trimethyl(trifluoromethyl)silane (2.5 mg, 17.5 μmol, 1 equiv.) was condensed onto this mixture. The mixture was allowed to warm up to −80 °C, the resulting solution was stirred for 1 hour, warmed to room temperature and left stirring overnight. Main reaction products identified by ^19^F NMR spectroscopy are *trans*‐[Au(CF_3_)F_2_(SIMes)] (**1**) and *cis*‐[Au(CF_3_)_2_F(SIMes)] (**2**), HCF_3_ and *trans*‐[AuClF_2_(SIMes)] are formed as by‐products (see Figures S9–S15). Single crystals of **1** suitable for X‐ray diffraction were obtained by slow vapor diffusion of *n*‐pentane into a dichloromethane solution at 5 °C.

*trans*‐[Au(CF_3_)F_2_(SIMes)] (**1**): ^1^H NMR (400 MHz, CD_2_Cl_2_, 20 °C): *δ*=7.04 (s, 4 H, *meta*‐CH), 4.12 (s, 4 H, NCH_2_CH_2_N), 2.33 (s, 18 H, *ortho*‐CH_3_ + *para*‐CH_3_) ppm. ^13^C NMR (101 MHz, CD_2_Cl_2_, 20 °C): *δ*=192.3 (s, NCN carbene), 139.7 (s, C_Ar_), 136.3 (s, C_Ar_), 132.5 (s, C_Ar_), 129.6 (s, C_Ar_), 51.4 (s, NCH_2_CH_2_N), 20.6 (s, CH_3_), 16.8 (s, CH_3_) ppm. ^19^F NMR (377 MHz, CD_2_Cl_2_, 20 °C): *δ*=−46.5 (t, 3F, *trans*‐CF_3_, ^3^ *J*(^19^F,^19^F)=18 Hz), −329.7 (q, 2F, *cis*‐F) ppm.

*cis*‐[Au(CF_3_)_2_F(SIMes)] (**2**): ^19^F NMR (377 MHz, CD_2_Cl_2_, 20 °C): *δ*=−23.9 (dq, 3F, *cis*‐CF_3_, ^3^ *J*(^19^F,^19^F)=57 Hz, ^4^ *J*(^19^F,^19^F)=7 Hz), −41.2 (dq, 3F, *trans*‐CF_3_, ^3^ *J*(^19^F,^19^F)=14 Hz), −254.0 (qq, 1F, *cis*‐F) ppm.

**Trifluoromethylation of [AuF_3_(SIMes)] in tetrahydrofuran**: In a typical experiment, CsF (2.7 mg, 17.8 μmol, 1 equiv.) was dissolved in tetrahydrofuran (1 mL) and at −196 °C, trimethyl(trifluoromethyl)silane (2.5 mg, 17.5 μmol, 1 equiv.) was added. The mixture was allowed to warm up to −80 °C and the resulting solution was stirred for 30 minutes. Thereafter, a solution of [AuF_3_(SIMes)] (10 mg, 17.8 μmol, 1 equiv.) in tetrahydrofuran (1 mL) was added and the resulting mixture was stirred for 1 hour at −80 °C, warmed to room temperature and left stirring overnight. Main reaction products identified by ^19^F NMR spectroscopy are *cis*‐[Au(CF_3_)_2_F(SIMes)] (**2**) and [Au(CF_3_)_3_(SIMes)] (**3**), HCF_3_ and [Au(CF_3_)_4_]^−^ are formed as by‐products (Supporting Information, Figures S16 and S17).

*cis*‐[Au(CF_3_)_2_F(SIMes)] (**2**): ^19^F NMR (377 MHz, ext. (CD_3_)_2_CO, 20 °C): *δ*=−24.1 (dq, 3F, *cis*‐CF_3_, ^3^ *J*(^19^F,^19^F)=57 Hz, ^4^ *J*(^19^F,^19^F)=7 Hz), −41.2 (dq, 3F, *trans*‐CF_3_, ^3^ *J*(^19^F,^19^F)=14 Hz), −253.6 (qq, 1F, *cis*‐F) ppm. [Au(CF_3_)_3_(SIMes)] (**3**): ^19^F NMR (377 MHz, ext. (CD_3_)_2_CO, 20 °C): *δ*=−31.3 (q, 6F, *cis*‐CF_3_, ^4^ *J*(^19^F,^19^F)=7 Hz), −34.1 (sep, 3F, *trans*‐CF_3_) ppm.

**Selective preparation of [Au(CF_3_)_3_(SIMes)] (3)**: [Au(CF_3_)_3_(NCMe)] (45 mg, 0.101 mmol, 1 equiv.) and SIMes (31 mg, 0.101 mmol, 1 equiv.) were dissolved in diethyl ether (1 mL) and stirred for 30 minutes at room temperature. The resulting suspension was filtered off, the colorless residue was washed with *n*‐hexane (2 mL) and residual solvent was removed under reduced pressure. The product was obtained as a colorless powder. Single crystals suitable for X‐ray diffraction were obtained from solutions of **3** in acetone, chloroform, dichloromethane or tetrahydrofuran, by layering them with *n*‐hexane. ^1^H NMR (400 MHz, CD_2_Cl_2_, 21 °C): *δ*=6.99 (s, 4 H, *meta*‐CH), 4.13 (s, 4 H, NCH_2_CH_2_N), 2.33 (s, 12 H, *ortho*‐CH_3_), 2.30 (s, 6 H, *para*‐CH_3_) ppm. ^13^C NMR (101 MHz, CD_2_Cl_2_, 21 °C): *δ*=191.4 (s, NCN carbene), 139.2 (s, C_Ar_), 135.4 (s, C_Ar_), 132.4 (s, C_Ar_), 129.6 (s, C_Ar_), 52.6 (s, NCH_2_CH_2_H), 20.4 (s, CH_3_), 17.5 (s, CH_3_) ppm. ^19^F NMR (377 MHz, CD_2_Cl_2_, 21 °C): *δ*=−31.5 (q, 6F, *cis*‐CF_3_, ^4^ *J*(^19^F,^19^F)=7 Hz), −34.3 (sep, 3F, *trans*‐CF_3_) ppm. IR (ATR, 25 °C, 4 cm^−1^): $\tilde{\nu }$ =3007 (w), 2960 (w), 2925 (w), 2869 (w), 1609 (m), 1495 (s), 1447 (m), 1380 (m), 1316 (w), 1272 (s), 1221 (w), 1159 (s, *ν*_as_(CF_3_)), 1109 (s, *ν*_as_(CF_3_)), 1069 (vs., *ν*_as_(CF_3_)_*trans*_), 1018 (s, *ν*_as_(CF_3_)_*cis*_), 949 (m), 916 (m), 886 (m), 862 (m), 740 (m), 631 (m), 579 (m), 565 (w), 532 (w), 502 (w), 434 (w) cm^−1^. FT‐Raman (25 °C, 30 mW, 2 cm^−1^): $\tilde{\nu }$ =3004 (m), 2927 (m), 2741 (w), 1823 (w), 1610 (m), 1494 (m), 1454 (m), 1387 (m), 1316 (m), 1225 (w), 1158 (w), 1089 (w), 1020 (w), 951 (w), 723 (m, *ν*_s_(CF_3_)), 581 (m), 566 (m), 517 (w), 475 (w), 340 (w), 260 (m, *ν*(AuC)), 233 (m, *ν*(AuC)), 86 (s) cm^−1^.

**Crystallographic data**: Deposition numbers 2001090, 2000997, 2000994, 2000995, and 2000996 (**1**, **3 a**, **3 b**, **3 c**, and **3 d**) contain the supplementary crystallographic data for this paper. These data are provided free of charge by the joint Cambridge Crystallographic Data Centre and Fachinformationszentrum Karlsruhe Access Structures service.

## Conflict of interest

The authors declare no conflict of interest.

## Supporting information

As a service to our authors and readers, this journal provides supporting information supplied by the authors. Such materials are peer reviewed and may be re‐organized for online delivery, but are not copy‐edited or typeset. Technical support issues arising from supporting information (other than missing files) should be addressed to the authors.

Supplementary
