# Supplementary material for: Trifluoromethylation of [AuF3(SIMes)]: Preparation and Characterization of [Au(CF3)xF3−x(SIMes)] (x=1–3) Complexes
Source: Chemistry. 2020 Oct 27;26(68):16089–97. doi: 10.1002/chem.202002940 (PMC7756667; doi:10.1002/chem.202002940)
Supplement: Supplementary file 1 — Supplementary [file CHEM-26-16089-s001.pdf]

Chemistry–A European Journal

Supporting Information

Trifluoromethylation of $[AuF_3(SIMes)]$: Preparation and Characterization of $[Au(CF_3)_xF_{3-x}(SIMes)]$ ($x = 1–3$) Complexes

Marlon Winter,[a] Niklas Limberg,[a] Mathias A. Ellwanger,[a] Alberto Pérez-Bitrián,[a, b] Karsten Sonnenberg,[a] Simon Steinhauer,[a] and Sebastian Riedel*[a]

Table of Contents

X-Ray Crystallography 2

Crystallographic Data 2

Molecular Structure of *trans*-[Au(CF_3)F_2(SIMes)] (1) in the Solid State 4

Molecular Structure of [Au(CF_3)$_3$(SIMes)]·0.5 CH_2Cl_2 (3a) in the Solid State 5

Molecular Structure of [Au(CF_3)$_3$(SIMes)]·0.5 $CHCl_3$ (3b) in the Solid State 5

Molecular Structure of [Au(CF_3)$_3$(SIMes)]·0.5 C_3H_6O (3c) in the Solid State 6

Molecular Structure of [Au(CF_3)$_3$(SIMes)]·0.5 C_5H_8O (3d) in the Solid State 6

NMR Spectroscopy 7

Summary of Products Identified by NMR Spectroscopy 7

NMR Spectra of the Reaction Between [AuF_3(SIMes)] and TMSCF_3 in DCM 8

NMR Spectra of the Reaction Between [AuF_3(SIMes)] and TMSCF_3 in THF 11

NMR Spectra of [Au(CF_3)$_3$(SIMes)] (3) 12

Vibrational Spectroscopy 14

IR and Raman Spectra of [Au(CF_3)$_3$(SIMes)] (3) 14

Quantum-Chemical Calculations 15

Coordinates of *trans*-[Au(CF_3)F_2(SIMes)] (1) on RI-B3LYP-D3/def2-TZVPP Level15

Coordinates of [Au(CF_3)$_3$(SIMes)] (3) on RI-B3LYP-D3/def2-TZVPP Level 16

Coordinates of SIMes on RI-B3LYP-D3/def2-TZVPP Level 17

Coordinates of [Au(CF_3)F_2] on RI-B3LYP-D3/def2-TZVPP Level 18

Coordinates of [Au(CF_3)$_3$] on RI-B3LYP-D3/def2-TZVPP Level 18

Literature 19

X-Ray Crystallography

Crystallographic Data

Table S1: Crystal data and refinement details for the analysis of the molecular structures in the solid state of *trans*-[Au(CF_3)F_2(SIMes)] (**1**), [Au(CF_3)$_3$(SIMes)]·0.5 CH_2Cl_2 (**3a**) and [Au(CF_3)$_3$(SIMes)]·0.5 $CHCl_3$ (**3b**). For **1**, the largest diffraction peak of 2.6 e Å^{-3} is close to the gold center and can be explained by the reduced precision of the crystal face determination due to large twin contributions and respective deviations of the numerical absorption correction.

	trans-[Au(CF_3)F_2(SIMes)] (**1**)	[Au(CF_3)$_3$(SIMes)]·0.5 CH_2Cl_2 (**3a**)	[Au(CF_3)$_3$(SIMes)]·0.5 $CHCl_3$ (**3b**)
Empirical formula	$C_{22}H_{26}AuF_5N_2$	$C_{49}H_{52}Au_2Cl_2F_{18}N_4$	$C_{49}H_{53}Au_2Cl_3F_{18}N_4$
Formula weight	610.41	1503.78	1540.23
Temperature/K	100.07	99.98	100.07
Crystal system	orthorhombic	monoclinic	monoclinic
Space group	*Pnma*	$P2_1/c$	$P2_1/c$
a/Å	15.183(6)	18.669(4)	19.0487(19)
b/Å	19.914(5)	9.174(2)	9.0163(8)
c/Å	7.540(3)	17.106(3)	17.3412(17)
α/°	90	90	90
β/°	90	116.201(7)	116.330(4)
γ/°	90	90	90
Volume/Å^3	2279.7(13)	2628.6(9)	2669.3(4)
Z	4	2	2
ρ_{calc}g/cm^3	1.779	1.900	1.916
μ/mm^{-1}	6.504	5.779	5.742
F(000)	1184.0	1456.0	1492.0
Crystal size/mm^3	0.546 × 0.252 × 0.195	0.12 × 0.11 × 0.1	0.45 × 0.39 × 0.32
Radiation	MoK$_\alpha$ (λ = 0.71073)	MoK$_\alpha$ (λ = 0.71073)	MoK$_\alpha$ (λ = 0.71073)
2Θ range for data collection/°	5.366 to 54.482	4.764 to 56.718	4.698 to 56.656
Index ranges	-19 ≤ h ≤ 19, -25 ≤ k ≤ 25, -9 ≤ l ≤ 9	-24 ≤ h ≤ 24, -12 ≤ k ≤ 12, -22 ≤ l ≤ 22	-25 ≤ h ≤ 25, -12 ≤ k ≤ 12, -23 ≤ l ≤ 23
Reflections collected	108341	106844	54535
Independent reflections	2618 [R_{int} = 0.0626, R_{sigma} = 0.0144]	6557 [R_{int} = 0.0574, R_{sigma} = 0.0195]	6623 [R_{int} = 0.0935, R_{sigma} = 0.0486]
Data/restraints/ parameters	2618/360/250	6557/7/351	6623/0/371
Goodness-of-fit on F^2	1.235	1.061	1.040
Final R indexes [I>=2σ (I)]	R_1 = 0.0329, wR_2 = 0.0726	R_1 = 0.0198, wR_2 = 0.0423	R_1 = 0.0300, wR_2 = 0.0644
Final R indexes [all data]	R_1 = 0.0430, wR_2 = 0.0814	R_1 = 0.0242, wR_2 = 0.0436	R_1 = 0.0391, wR_2 = 0.0679
Largest diff. peak/hole / e Å^{-3}	2.62/-1.94	1.53/-1.37	1.01/-1.85
CCDC deposition number	2001090	2000997	2000994

Table S2: Crystal data and refinement details for the analysis of the molecular structures in the solid state of [Au$(CF_3)_3$(SIMes)]·C_3H_6O (**3c**) and [Au$(CF_3)_3$(SIMes)]·C_5H_8O (**3d**).

	[Au$(CF_3)_3$(SIMes)]·0.5 C_3H_6O (**3c**)	[Au$(CF_3)_3$(SIMes)]·0.5 C_5H_8O (**3d**)
Empirical formula	$C_{51}H_{58}Au_2F_{18}N_4O$	$C_{52}H_{60}Au_2F_{18}N_4O$
Formula weight	1478.94	1492.97
Temperature/K	100.01	100.02
Crystal system	monoclinic	monoclinic
Space group	$P2_1/c$	$P2_1/c$
a/Å	18.8838(13)	19.016(4)
b/Å	9.0553(5)	9.0238(13)
c/Å	17.2002(11)	17.301(4)
α/°	90	90
β/°	116.683(2)	115.892(5)
γ/°	90	90
Volume/Å^3	2628.0(3)	2670.8(9)
Z	2	2
ρ_{calc}g/cm^3	1.869	1.856
μ/mm^{-1}	5.682	5.592
F(000)	1440.0	1456.0
Crystal size/mm^3	0.24 × 0.24 × 0.08	0.2 × 0.11 × 0.04
Radiation	MoK$_\alpha$ (λ = 0.71073)	MoK$_\alpha$ (λ = 0.71073)
2Θ range for data collection/°	4.736 to 56.638	4.71 to 54.902
Index ranges	-25 ≤ h ≤ 25, -12 ≤ k ≤ 12, -22 ≤ l ≤ 22	-24 ≤ h ≤ 24, -11 ≤ k ≤ 11, -22 ≤ l ≤ 22
Reflections collected	60698	74712
Independent reflections	6520 [R_{int} = 0.0665, R_{sigma} = 0.0326]	6097 [R_{int} = 0.1016, R_{sigma} = 0.0393]
Data/restraints/ parameters	6520/6/369	6097/0/376
Goodness-of-fit on F^2	1.046	1.032
Final R indexes [I>=2σ (I)]	R_1 = 0.0225, wR_2 = 0.0490	R_1 = 0.0281, wR_2 = 0.0464
Final R indexes [all data]	R_1 = 0.0298, wR_2 = 0.0514	R_1 = 0.0431, wR_2 = 0.0498
Largest diff. peak/hole / e Å^{-3}	0.65/-1.73	1.02/-0.89
CCDC deposition number	2000995	2000996

Molecular Structure of *trans*-[Au(CF_3)F_2(SIMes)] (1) in the Solid State

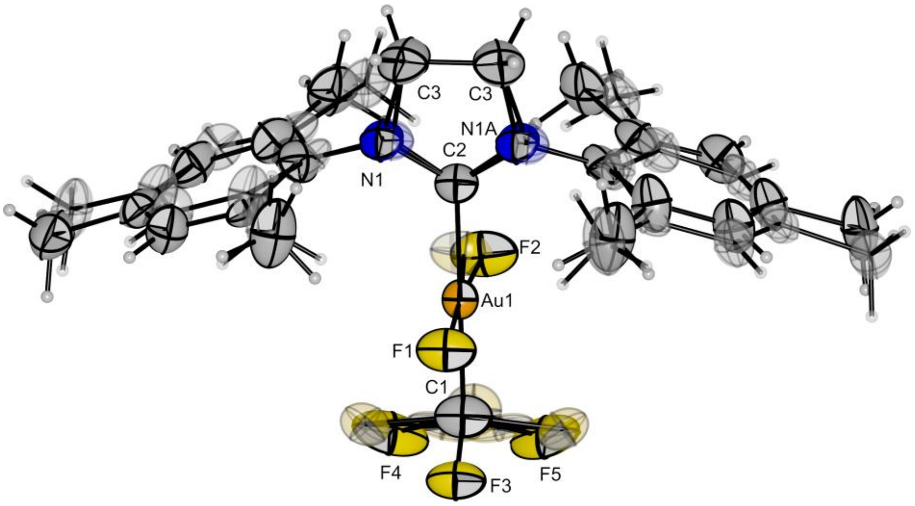

Figure S1: Molecular structure of *trans*-[Au(CF_3)F_2(SIMes)] (**1**) in the solid state. The second position of disordered atoms in the CF_3 and the SIMes ligand is shown as transparent ellipsoids. Thermal ellipsoids are set at 50 % probability. Bond lengths [pm] to the central gold atom: 193.2(5) (F1-Au1), 193.2(10) (F2-Au1), 203.6(10) (C1-Au1), 203.5(9) (C2-Au1).

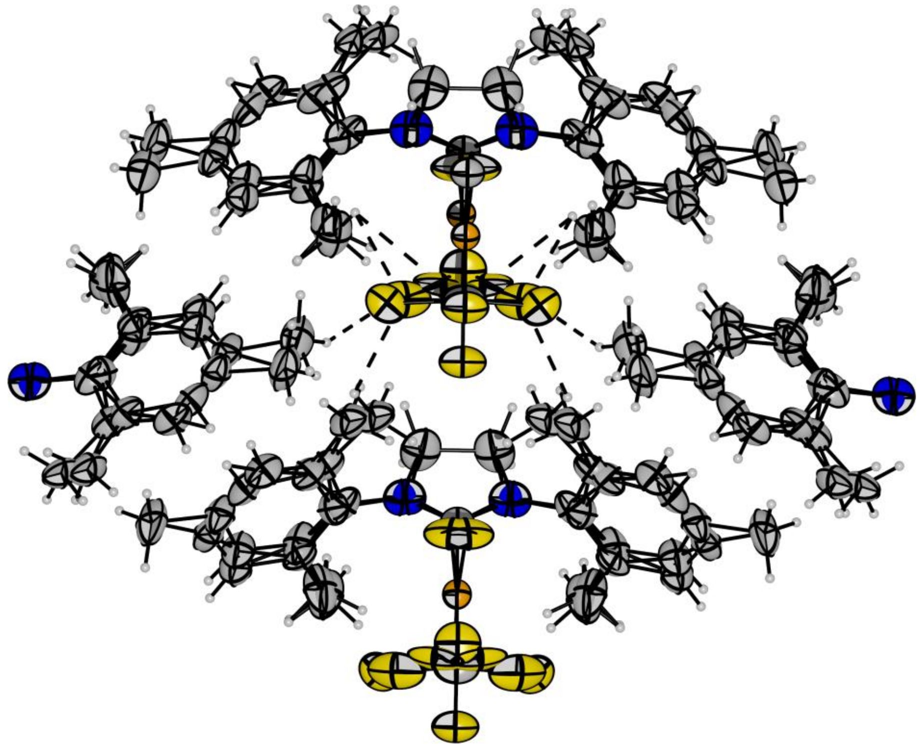

Figure S2: Crystal packing via intermolecular H-F contacts (*r*[C(H)-F] < 300 pm; dashed bonds), shown exemplarily for one entity of *trans*-[Au(CF_3)F_2(SIMes)] (**1**), including disorders. Thermal ellipsoids are set at 50 % probability.

Molecular Structure of $[Au(CF_3)_3(SIMes)]\cdot 0.5\ CH_2Cl_2$ (3a) in the Solid State

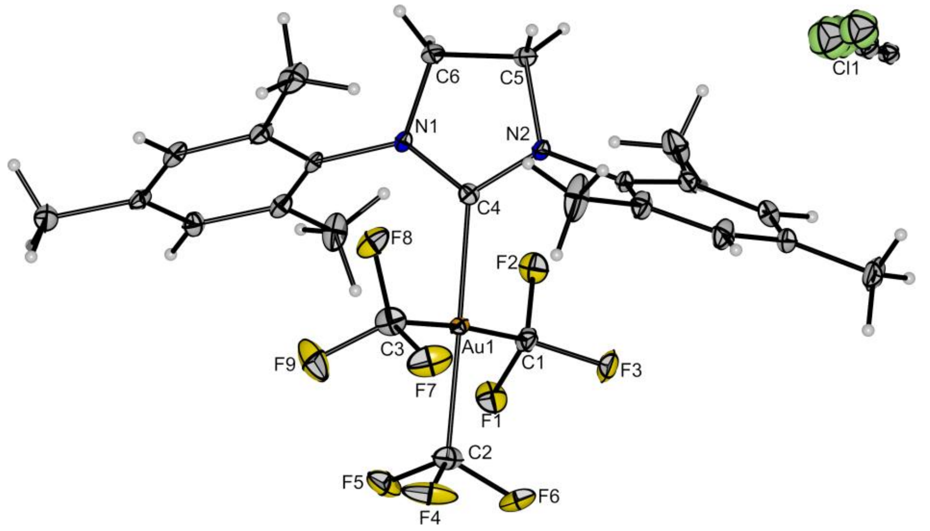

Figure S3: Molecular structure of $[Au(CF_3)_3(SIMes)]\cdot 0.5\ CH_2Cl_2$ (**3a**) in the solid state. Thermal ellipsoids are set at 50 % probability. Bond lengths [pm] to the central gold atom: 208.6(3) (C1-Au1), 207.8(3) (C2-Au1), 208.3(3) (C3-Au1), 208.1(2) (C4-Au1).

Molecular Structure of $[Au(CF_3)_3(SIMes)]\cdot 0.5\ CHCl_3$ (3b) in the Solid State

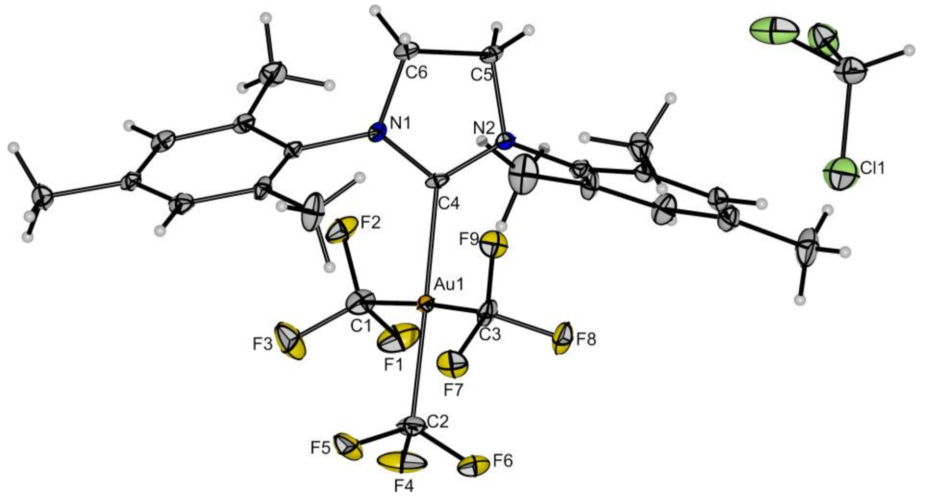

Figure S4: Molecular structure of $[Au(CF_3)_3(SIMes)]\cdot 0.5\ CHCl_3$ (**3b**) in the solid state. Thermal ellipsoids are set at 50 % probability. Bond lengths [pm] to the central gold atom: 208.9(4) (C1-Au1), 207.7(4) (C2-Au1), 209.4(4) (C3-Au1), 208.5(3) (C4-Au1).

Molecular Structure of $[Au(CF_3)_3(SIMes)]\cdot 0.5\ C_3H_6O$ (3c) in the Solid State

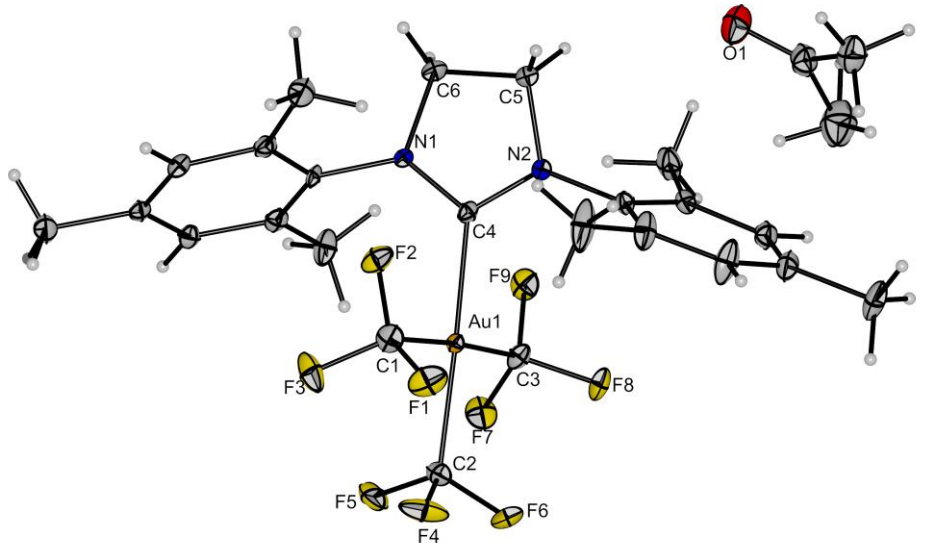

Figure S5: Molecular structure of $[Au(CF_3)_3(SIMes)]\cdot 0.5\ C_3H_6O$ (**3c**) in the solid state. Thermal ellipsoids are set at 50 % probability. Bond lengths [pm] to the central gold atom: 208.5(3) (C1-Au1), 207.3(3) (C2-Au1), 208.7(3) (C3-Au1), 207.7(3) (C4-Au1).

Molecular Structure of $[Au(CF_3)_3(SIMes)]\cdot 0.5\ C_5H_8O$ (3d) in the Solid State

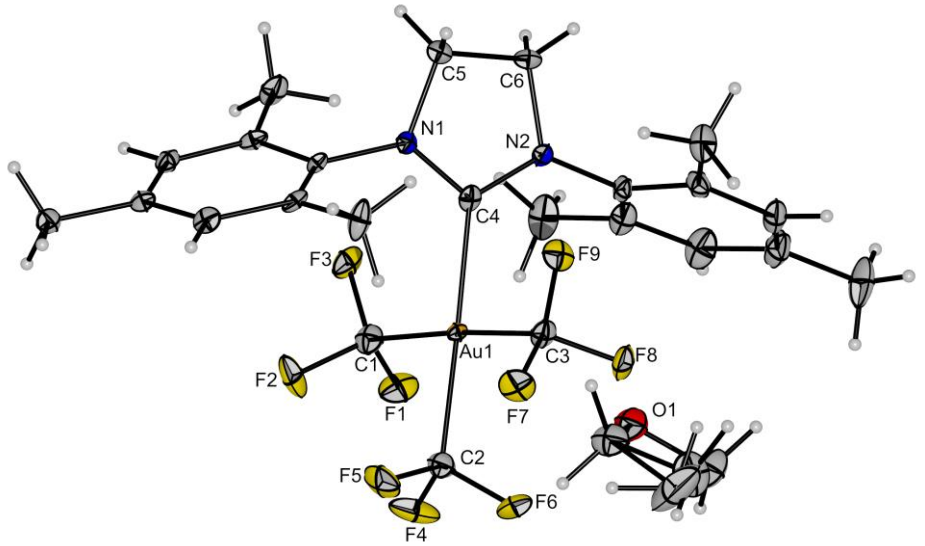

Figure S6: Molecular structure of $[Au(CF_3)_3(SIMes)]\cdot 0.5\ C_5H_8O$ (**3d**) in the solid state. Thermal ellipsoids are set at 50 % probability. Bond lengths [pm] to the central gold atom: 208.8(4) (C1-Au1), 207.5(4) (C2-Au1), 208.4(4) (C3-Au1), 207.4(3) (C4-Au1).

NMR Spectroscopy

Summary of Products Identified by NMR Spectroscopy

Figure S7 shows the products that have been identified by ^{19}F NMR spectroscopy. Their assignment in the following spectra is done using the numbers written in bold.[1] For compounds **1 - 3**, which incorporate different fluorine-containing groups, the different groups will be denoted by F for fluorine atoms and CF_3 for trifluoromethyl groups. Furthermore, c for ligands *cis* to the SIMes ligand (cf. Figure S8) and t for *trans* to SIMes are used as subscripts in case of chemically inequivalent ligands of the same type (compound **2** and **3**). Figure S8 depicts the structure of SIMes denoting the chemically inequivalent hydrogen and carbon atoms, which can be distinguished in the ^{1}H and ^{13}C NMR spectra that are shown below.

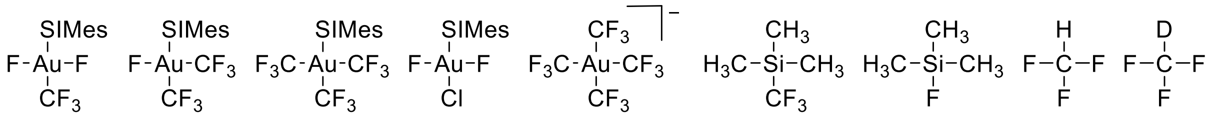

1 **2** **3** **4** **5** **6** **7** **8** **9**

Figure S7: List of products that have been detected in the ^{19}F NMR spectra of the reactions that are presented in this work. The numbers in bold are used for their assignment in the following ^{19}F NMR spectra.

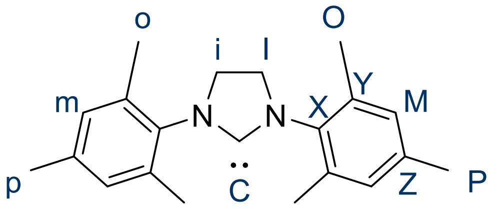

Figure S8: Structure of the NHC 1,3-bis(2,4,6-trimethylphenyl)-4,5-dihydroimidazol-2-ylidene (SIMes), which is present in products **1**-**4** (cf. Figure S7). The positions of chemically inequivalent hydrogen atoms are denoted in blue small letters, those of the chemically inequivalent carbon atoms by blue capital letters. C = carbene, I = imidazolidine, M = *meta* C, O = *ortho* CH_3, P = *para* CH_3, X = *ipso* C, Y = *ortho* C, Z = *para* C, i = imidazolidine H, m = *meta* H, o = *ortho* H, p = *para* H.

NMR Spectra of the Reaction Between [AuF_3(SIMes)] and $TMSCF_3$ in DCM

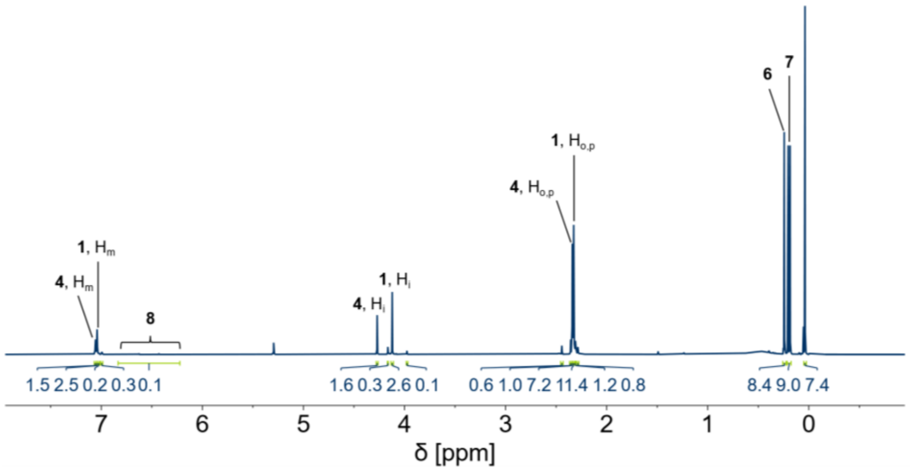

Figure S9: ^{1}H NMR spectrum (400 MHz, CD_2Cl_2, 22 °C) of the reaction between 1 eq. of [AuF_3(SIMes)] and 0.5 eq. of $TMSCF_3$ in DCM including assignments to the compounds shown in Figure S7. The integrals were referenced to TMSF (**7**) with an integral of 9.0.

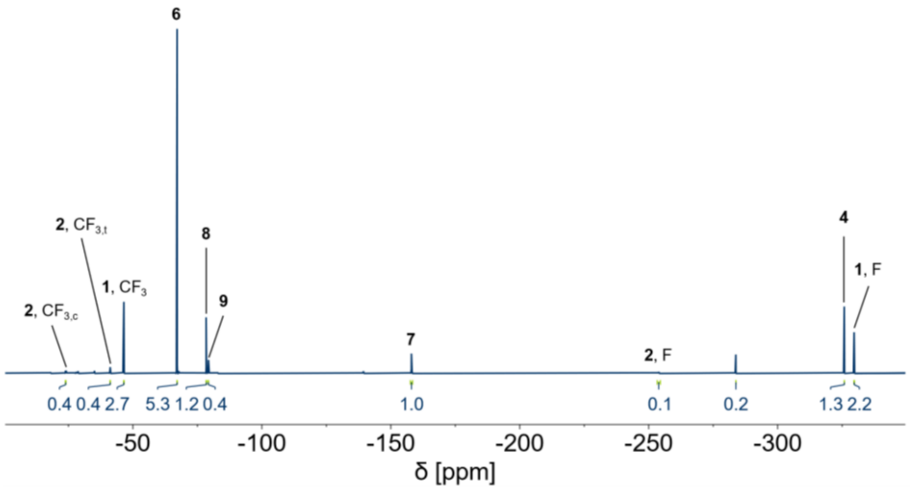

Figure S10: ^{19}F NMR spectrum (377 MHz, CD_2Cl_2, 22 °C) of the reaction between 1 eq. of [AuF_3(SIMes)] and 0.5 eq. of $TMSCF_3$ in DCM including assignments to the compounds shown in Figure S7. The integrals were referenced to TMSF (**7**) with an integral of 1.0.

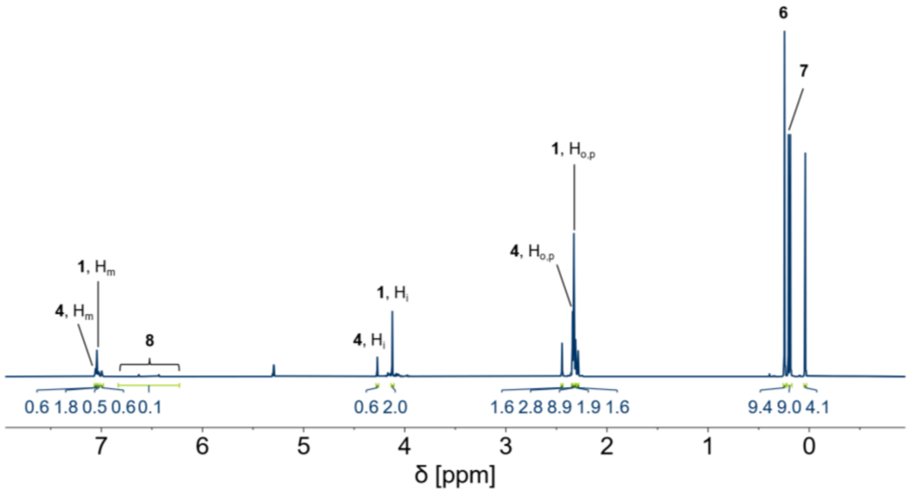

Figure S11: ^{1}H NMR spectrum (400 MHz, CD_2Cl_2, 20 °C) of the reaction between 1 eq. of [AuF_3(SIMes)] and 1 eq. of $TMSCF_3$ in DCM including assignments to the compounds shown in Figure S7. The integrals were referenced to TMSF (**7**) with an integral of 9.0.

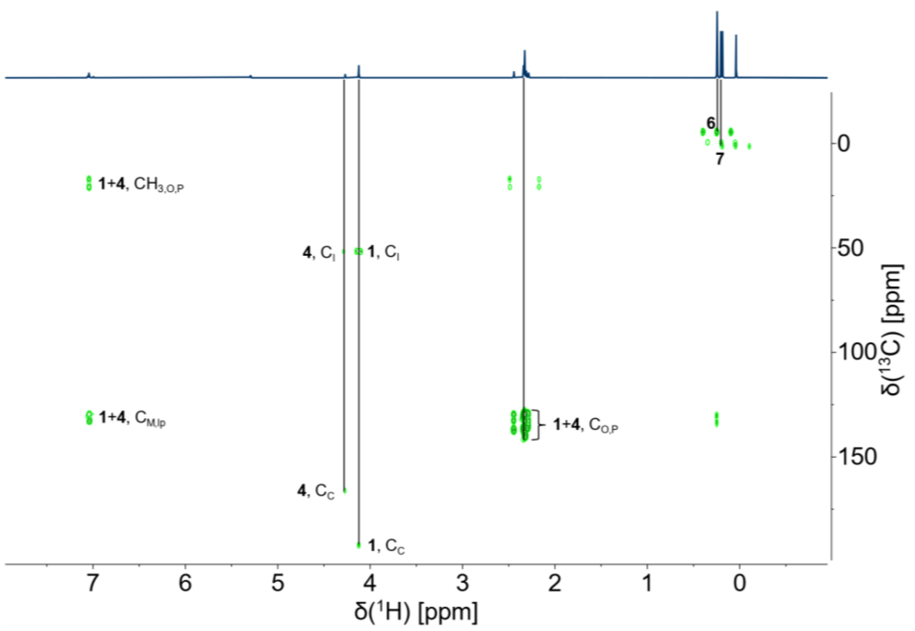

Figure S12: ^{1}H,^{13}C HMBC NMR spectrum (400 MHz, CD_2Cl_2, 20 °C) of the reaction between 1 eq. of [AuF_3(SIMes)] and 1 eq. of $TMSCF_3$ in DCM including assignments to the compounds shown in Figure S7.

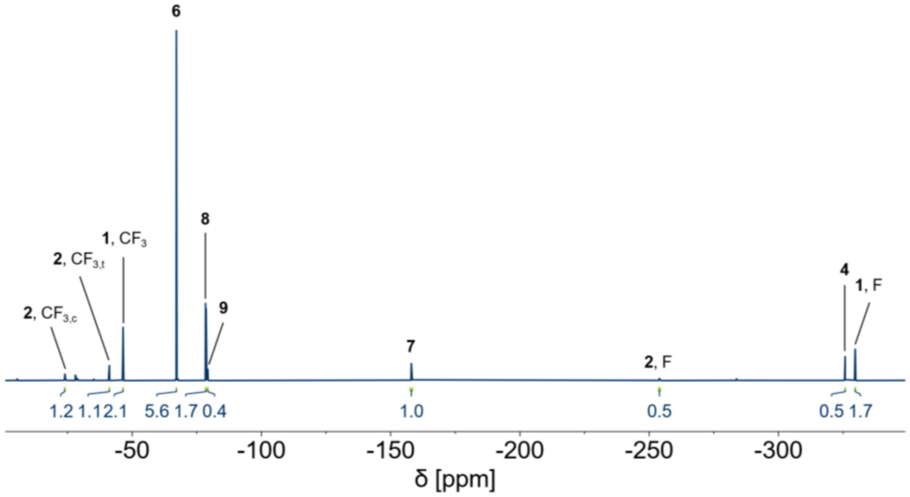

Figure S13: ^{19}F NMR spectrum (377 MHz, CD_2Cl_2, 20 °C) of the reaction between 1 eq. of [AuF_3(SIMes)] and 1 eq. of $TMSCF_3$ in DCM including assignments to the compounds shown in Figure S7. The integrals were referenced to TMSF (**7**) with an integral of 1.0.

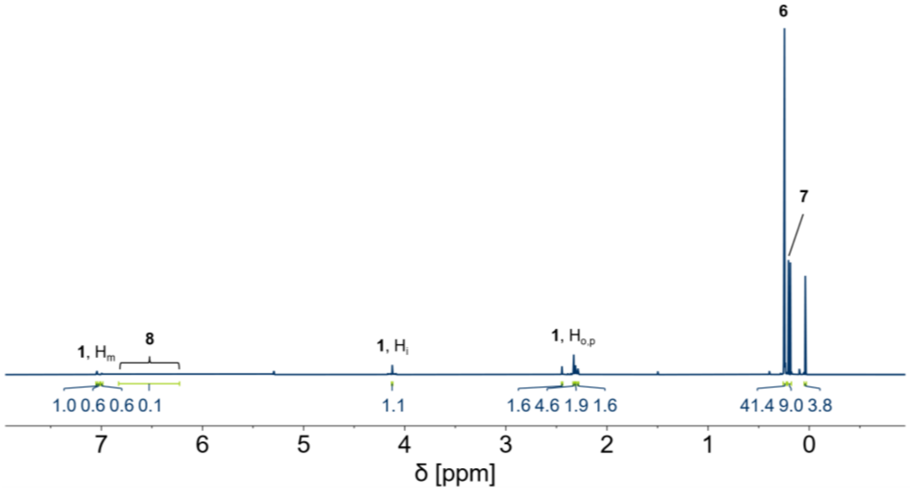

Figure S14: ^{1}H NMR spectrum (400 MHz, CD_2Cl_2, 21 °C) of the reaction between 1 eq. of [AuF_3(SIMes)] and 5 eq. of $TMSCF_3$ in DCM including assignments to the compounds shown in Figure S7. The integrals were referenced to TMSF (**7**) with an integral of 9.0.

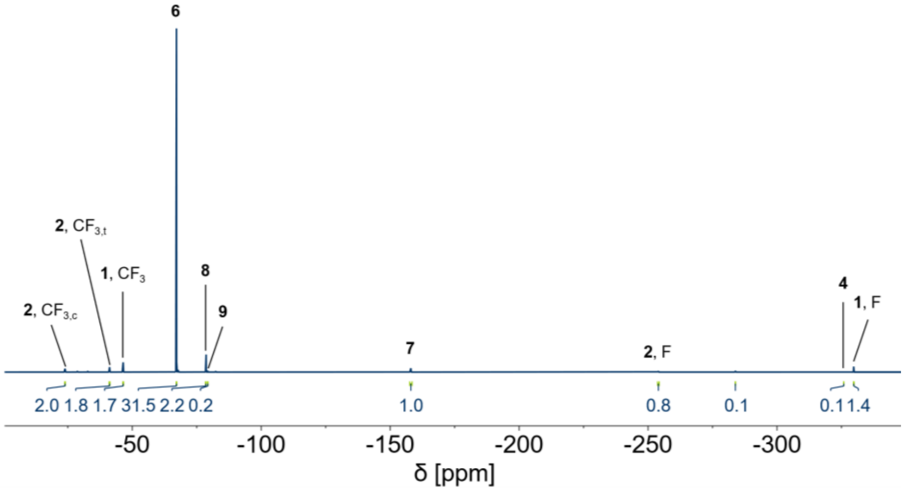

Figure S15: ^{19}F NMR spectrum (377 MHz, CD_2Cl_2, 21 °C) of the reaction between 1 eq. of [AuF_3(SIMes)] and 5 eq. of $TMSCF_3$ in DCM including assignments to the compounds shown in Figure S7. The integrals were referenced to TMSF (**7**) with an integral of 1.0.

NMR Spectra of the Reaction Between [AuF_3(SIMes)] and $TMSCF_3$ in THF

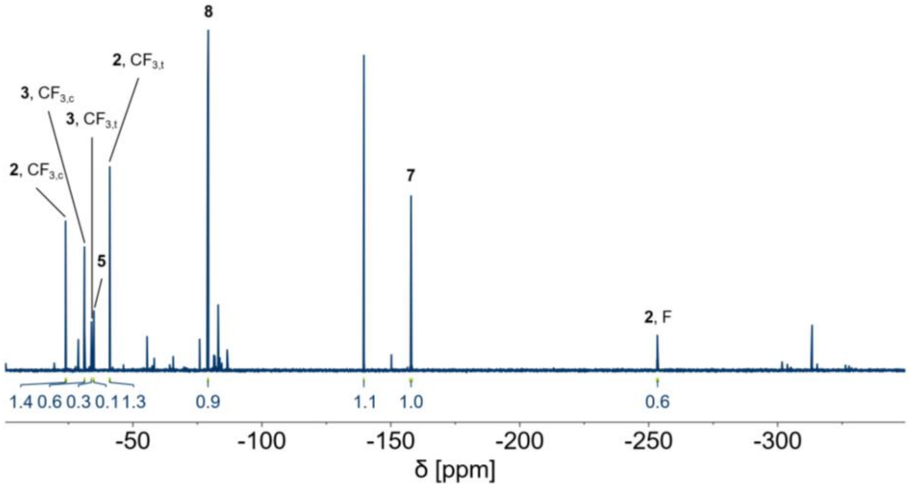

Figure S16: ^{19}F NMR spectrum (377 MHz, ext.$(CD_3)_2CO$, 20 °C) of the reaction between 1 eq. of [AuF_3(SIMes)] and 0.5 eq. of $TMSCF_3$ in THF including assignments to the compounds shown in Figure S7. The integrals were referenced to TMSF (**7**) with an integral of 1.0.

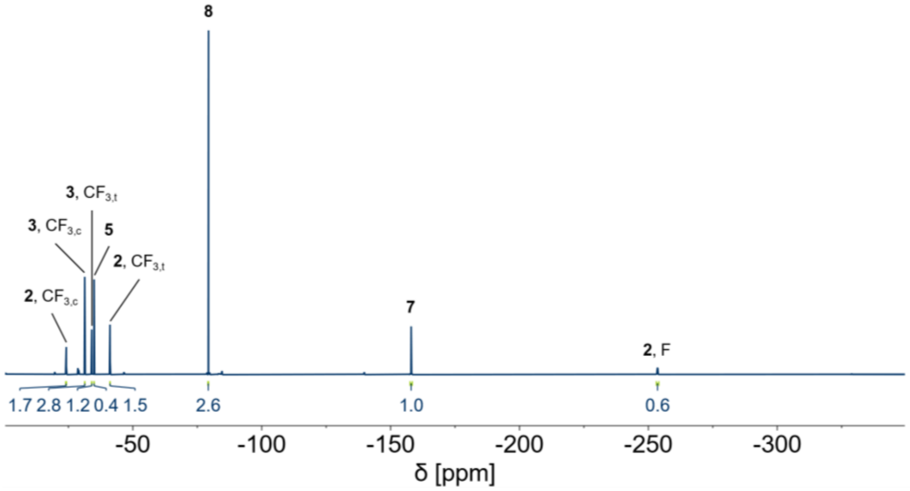

Figure S17: ^{19}F NMR spectrum (377 MHz, ext.$(CD_3)_2CO$, 21 °C) of the reaction between 1 eq. of [AuF_3(SIMes)] and 1 eq. of $TMSCF_3$ in THF including assignments to the compounds shown in Figure S7. The integrals were referenced to TMSF (**7**) with an integral of 1.0.

NMR Spectra of [Au(CF_3)$_3$(SIMes)] (3)

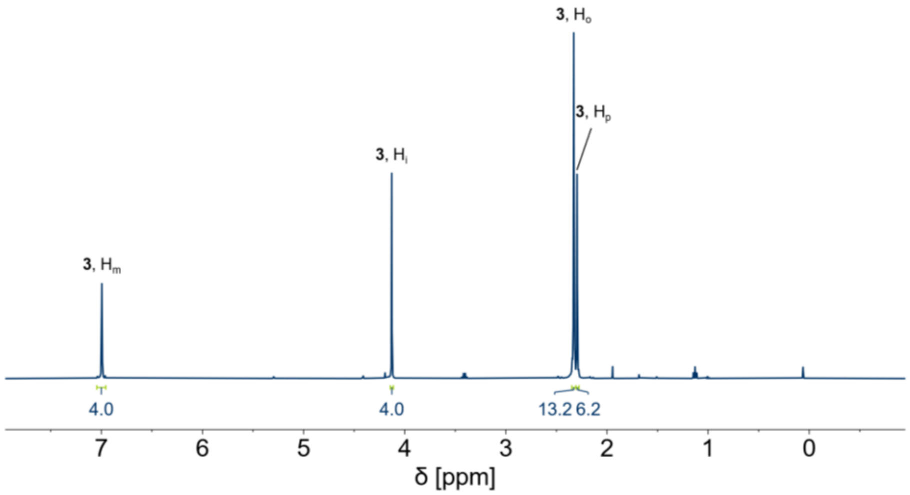

Figure S18: ^{1}H NMR spectrum (400 MHz, CD_2Cl_2, 21 °C) of [Au(CF_3)$_3$(SIMes)] (**3**). The integrals were referenced to the signal of the hydrogen atoms in *meta* position of the SIMes ligand in compound **3** with an integral of 4.0.

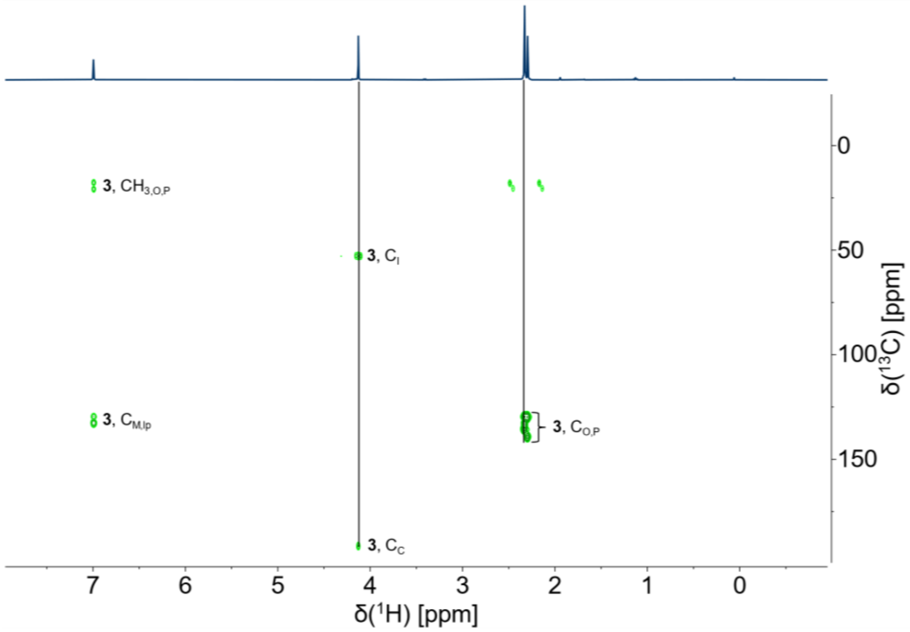

Figure S19: ^{1}H,^{13}C HMBC NMR spectrum (400 MHz, CD_2Cl_2, 21 °C) of [Au(CF_3)$_3$(SIMes)] (**3**).

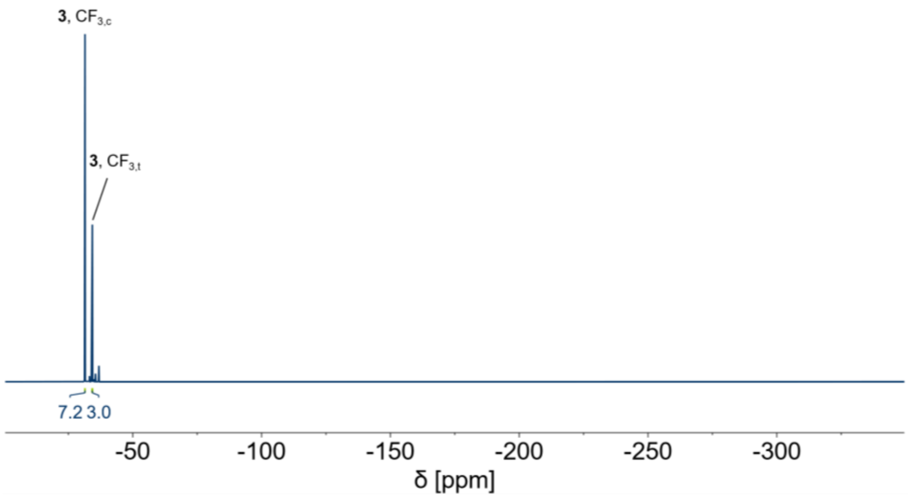

Figure S20: ^{19}F NMR spectrum (377 MHz, CD_2Cl_2, 21 °C) of $[Au(CF_3)_3(SIMes)]$ (**3**). The integrals were referenced to the signal of the CF_3 group *trans* to the SIMes ligand of compound **3** with an integral of 3.0.

Vibrational Spectroscopy

IR and Raman Spectra of [Au(CF_3)$_3$(SIMes)] (3)

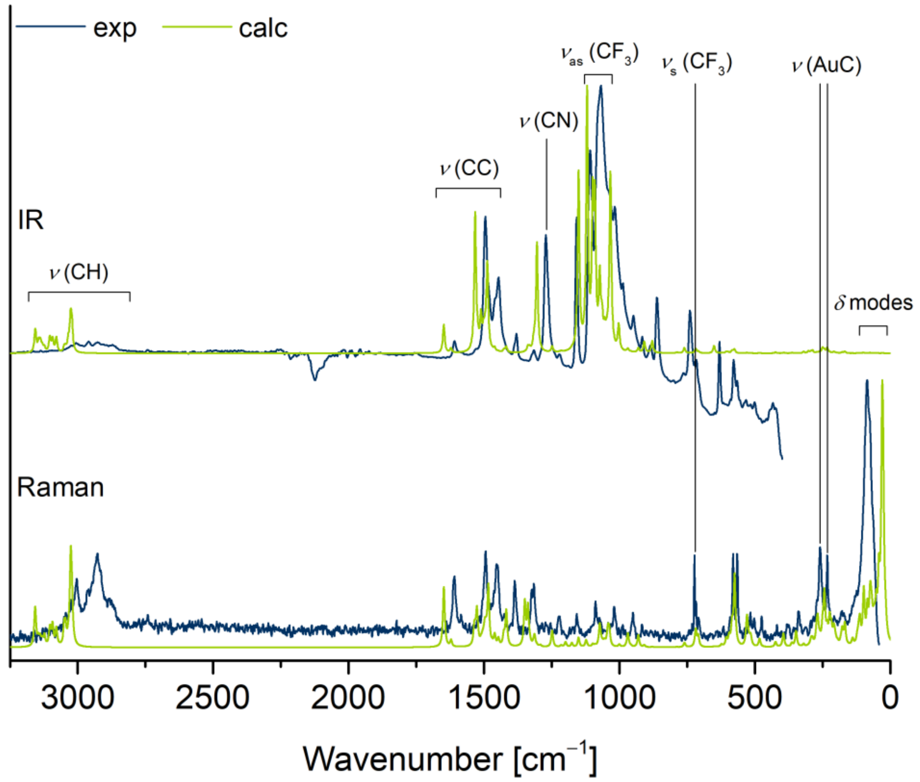

Figure S21: IR (top) and Raman (bottom) spectra of [Au(CF_3)$_3$(SIMes)] (**3**) taken at room temperature (blue lines) compared to the respective calculated spectra at the RI-B3LYP-D3/def2-TZVPP level of theory (green lines) including assignments of the most pronounced bands. The shift of the baseline in the experimental IR spectrum is due to pressure changes inside the glovebox during the measurement.

Quantum-Chemical Calculations

Coordinates of *trans*-[Au(CF$_3$)F$_2$(SIMes)] (1) on RI-B3LYP-D3/def2-TZVPP Level

xyz-Coordinates [Å] of the optimized minimum structure of *trans*-[Au(CF$_3$)F$_2$(SIMes)].

56

N	0.50168306269757	-1.99122569872415	-2.12674790065973	7
N	-0.53866790244528	1.99417766254798	-2.11469340933680	7
C	-0.00050843175022	0.00186820955035	-0.69750370189893	6
C	-0.41745073832938	1.41036752815574	-4.84178197020148	6
C	0.30920768434604	-1.40956954244729	-4.85017826708545	6
C	1.15076290440180	-4.44868491982770	-1.19161674601552	6
C	3.69680902373326	-5.02510470133836	-0.77870610839879	6
C	4.27987890025913	-7.42781924324619	0.12162929786223	6
C	2.41037217375334	-9.21318692508954	0.62293583024689	6
C	-0.10482084694442	-8.55486521803787	0.20622738506014	6
C	-0.78527893213047	-6.17728673257122	-0.69280731603043	6
C	5.72547658674054	-3.07525258524604	-1.19009384574451	6
C	3.08764063371387	-11.76942324496073	1.67457684340703	6
C	-3.51621593647022	-5.45222279185808	-1.01419838874388	6
C	-1.16068880760291	4.45295638494358	-1.16476069667449	6
C	-3.69317731372968	5.02926584373529	-0.67540512080850	6
C	-4.24924024968166	7.43322503302532	0.23849699837034	6
C	-2.36608185227799	9.22007468499799	0.67940700994380	6
C	0.13550329546140	8.56206618015286	0.18682557520825	6
C	0.78899735012571	6.18309053372043	-0.72841151140417	6
C	-5.73304339504958	3.07839485500967	-1.02095037577768	6
C	-3.01263215633849	11.77748010556034	1.74737652670983	6
C	3.50894168664886	5.45807515581372	-1.13275172272562	6
H	0.98896647981425	2.60435420968756	-5.75613772800017	1
H	-2.24332304001492	1.77169399662858	-5.72201321239048	1
H	-1.12089413125999	-2.60434598167944	-5.72605813196994	1
H	2.11115699132340	-1.77168830331241	-5.77801725045623	1
H	6.24164417562138	-7.90654629278944	0.45957294659886	1
H	-1.57915955581735	-9.91669800343994	0.61059318974417	1
H	5.63853375898020	-2.25832345242663	-3.08302502977390	1
H	7.59384756922415	-3.90135180420294	-0.94649335254790	1
H	5.51762602551024	-1.53057131868024	0.16246119657307	1
H	3.10976011847519	-11.71950917546080	3.73943773670095	1
H	4.95698100884999	-12.37100843054102	1.05153208233453	1
H	1.72339125010672	-13.20500141350158	1.10668902638181	1
H	-4.03947812349837	-4.00058604824113	0.35626788290813	1
H	-4.74271223277539	-7.07946437976664	-0.73082199491879	1
H	-3.89704889962521	-4.69395466263632	-2.89511403340183	1
H	-6.19983924845516	7.91160490899969	0.63613996653767	1
H	1.62092370435065	9.92535441769685	0.54297223967289	1
H	-5.69351387952116	2.24614414944257	-2.90871911930926	1
H	-7.59403427712362	3.90834387154591	-0.73732370480068	1
H	-5.49378636564970	1.54417258388353	0.33839779749260	1
H	-4.89264842551322	12.38569356597990	1.16399247617064	1
H	-1.65593147579187	13.20925990046959	1.15260757736794	1
H	-2.99257183574096	11.72525263030292	3.81219959633269	1
H	4.06892058409079	3.99595282121731	0.21169335112899	1
H	4.74476275446736	7.08226005732116	-0.87326102767513	1
H	3.83517907811662	4.71344351228417	-3.02939227712221	1
Au	0.03955147933681	-0.00025138550749	3.28530808048718	79
F	-3.54478598360196	-0.89488101465213	3.33514697724602	9
F	3.62067770578321	0.89673633616878	3.20885389990659	9
C	-0.00251992748870	-0.02170267647272	7.22137731499415	6
F	-0.75109191128513	-2.29494275877852	8.12617171691594	9
F	-1.64535973597762	1.71448957593359	8.13705313461235	9
F	2.25930962595822	0.47966999066129	8.27104028695687	9

E_{tot} = –1598.31784268855 H

Coordinates of [Au(CF_3)$_3$(SIMes)] (3) on RI-B3LYP-D3/def2-TZVPP Level

xyz-Coordinates [Å] of the optimized minimum structure of [Au(CF_3)$_3$(SIMes)].

62

N	0.42943905835783	-1.96054738023150	-2.30572899438743	7
N	-0.67536248845823	2.01380214861782	-2.24376125395314	7
C	-0.05821176523399	0.02332027581897	-0.82579031109381	6
C	-0.33170287743976	1.47556922051452	-4.96181679450861	6
C	-0.15897511694741	-1.40463209339656	-4.97802368155276	6
C	1.25086243073304	-4.42152361215396	-1.51944955036556	6
C	3.72282257370981	-5.17620639348333	-2.12342328281884	6
C	4.54057279738046	-7.52971137994382	-1.29300593667418	6
C	2.98534662749340	-9.14206498041803	0.08917828997449	6
C	0.52275439415313	-8.37165771081662	0.57306220964121	6
C	-0.40014005548471	-6.03639521742287	-0.22567580215325	6
C	5.49376605056994	-3.55888680104508	-3.65870808921555	6
C	3.96705164132246	-11.62478177115899	1.07010876320245	6
C	-3.11230616441070	-5.37561651306191	0.32344510166653	6
C	-1.33896728907114	4.48943310712141	-1.36693774916851	6
C	-3.82339710688553	5.34278777186383	-1.73769454285146	6
C	-4.46714543513667	7.72023986489721	-0.82452229275919	6
C	-2.72925998066087	9.25913069912863	0.41512091112369	6
C	-0.26424303329753	8.38935827953514	0.66820901992457	6
C	0.48719352036343	6.02684986602159	-0.22242413290507	6
C	-5.79004941288518	3.81101477718016	-3.11418093591607	6
C	-3.51602731285537	11.77226492074174	1.49016791467428	6
C	3.20943932982291	5.25276383981805	0.06869338447574	6
H	1.40620597762873	2.38295120479088	-5.61132644079736	1
H	-1.90440992470172	2.20416392491663	-6.06084365245889	1
H	-1.94984672789450	-2.30654127477299	-5.47164061222460	1
H	1.30552824340536	-2.12650914041006	-6.22110157219446	1
H	6.45934723339690	-8.10760831663938	-1.71536505377037	1
H	-0.73637961854007	-9.61794278707549	1.60016402168918	1
H	5.42116852189644	-4.09029917446694	-5.65498414054972	1
H	7.43392512307407	-3.81834204771115	-3.02439914838259	1
H	5.05841659961981	-1.55883573399346	-3.50651078388328	1
H	4.90947566078509	-11.35706915151400	2.88841104544557	1
H	5.34163833931879	-12.45662190694973	-0.22034065316263	1
H	2.44340620172536	-12.98114049405513	1.35589616292112	1
H	-3.36227811750808	-4.81085612812854	2.29053713183204	1
H	-4.31519150735771	-7.01880566742463	0.01111215475341	1
H	-3.80937506399038	-3.83529896048284	-0.83946475093177	1
H	-6.39203239572923	8.37615026170602	-1.06647371760398	1
H	1.13240544317937	9.57931511876093	1.57788541659890	1
H	-5.39694182428749	1.79827539882528	-3.05919069480241	1
H	-5.91397900338623	4.39857422785116	-5.09217143987651	1
H	-7.64453226188016	4.09957171121638	-2.27000512033550	1
H	-4.97861411331775	12.66049246063270	0.34204514311908	1
H	-1.92184760769221	13.06981960910173	1.62531547653890	1
H	-4.28436809270072	11.53229826625171	3.39230311911736	1
H	3.62038184401762	4.67771665441428	2.00342309668617	1
H	4.44547847795731	6.84286394160396	-0.36588749472411	1
H	3.73107989541632	3.68232616977397	-1.14685842720923	1
Au	0.02778432612641	-0.07750485160451	3.17158222885068	79
C	-0.04158315522507	-0.40729901054580	7.15088102359845	6
F	-0.98440090196304	-2.70659650220305	7.80460309690130	9
F	-1.53780440065124	1.34406388721716	8.26700951304712	9
F	2.22076506253274	-0.22408014304978	8.31343920492982	9
C	-3.96605239688926	0.51303213683859	3.33754166457242	6
F	-5.18897031983288	0.14288354148524	1.07602773824589	9
F	-4.54093210892378	2.91450291347810	4.01851409313150	9
F	-5.18622165196947	-1.02266393351948	4.98177694880986	9
C	4.02855936470347	-0.57793634414143	3.20940600897762	6
F	5.13578788750245	-0.46980707384836	0.86520837628902	9
F	5.25741423757663	1.21802629525339	4.56938983949151	9
F	4.73353236943844	-2.83977999970802	4.17724895300116	9

$E_{tot} = -2073.93400595089$ H

Coordinates of SIMes on RI-B3LYP-D3/def2-TZVPP Level

xyz-Coordinates [Å] of the optimized minimum structure of SIMes.

49

N	-0.2150429	-1.0038805	-0.6330203	7
N	0.5837386	0.9751496	-0.4064118	7
C	-0.0719539	0.0014631	0.2444842	6
C	0.8385285	0.6949789	-1.8417560	6
C	0.4477449	-0.7800335	-1.9421970	6
C	-0.7856218	-2.2721692	-0.3190063	6
C	-0.0385732	-3.2073218	0.4077555	6
C	-0.6104343	-4.4478597	0.6791185	6
C	-1.8931269	-4.7766786	0.2452920	6
C	-2.6142920	-3.8209128	-0.4653608	6
C	-2.0821033	-2.5658033	-0.7552681	6
C	1.3393066	-2.8644599	0.9073603	6
C	-2.4747961	-6.1390938	0.5211980	6
C	-2.8952840	-1.5374977	-1.4960017	6
C	0.8557706	2.2651506	0.1366482	6
C	-0.1739412	3.2100941	0.2268971	6
C	0.1247351	4.4711309	0.7365881	6
C	1.4113354	4.8110903	1.1501521	6
C	2.4103720	3.8458568	1.0579284	6
C	2.1542800	2.5700013	0.5582962	6
C	-1.5761466	2.8569185	-0.1912927	6
C	1.7144611	6.1949357	1.6636683	6
C	3.2437870	1.5321524	0.5019681	6
H	1.8801328	0.8811831	-2.1003087	1
H	0.2139050	1.3397770	-2.4654913	1
H	-0.2314963	-0.9884222	-2.7678091	1
H	1.3138330	-1.4396500	-2.0408552	1
H	-0.0407484	-5.1743174	1.2474763	1
H	-3.6219153	-4.0513538	-0.7922432	1
H	1.9933223	-2.5468696	0.0925220	1
H	1.8014010	-3.7197891	1.3986163	1
H	1.2941511	-2.0350330	1.6148786	1
H	-2.1823686	-6.8520485	-0.2545999	1
H	-3.5645924	-6.1106487	0.5442619	1
H	-2.1254267	-6.5358262	1.4752073	1
H	-2.8628010	-0.5744440	-0.9856509	1
H	-3.9350776	-1.8506197	-1.5815034	1
H	-2.5161842	-1.3763030	-2.5083906	1
H	-0.6687782	5.2055365	0.8160643	1
H	3.4125879	4.0857177	1.3944114	1
H	-1.6085195	2.4982410	-1.2222596	1
H	-2.2369256	3.7192117	-0.1120906	1
H	-1.9704050	2.0535938	0.4330156	1
H	1.9188487	6.8810521	0.8371814	1
H	2.5894660	6.1968919	2.3141800	1
H	0.8735542	6.6045372	2.2248156	1
H	2.9099321	0.5937321	0.9459461	1
H	4.1326910	1.8708916	1.0326363	1
H	3.5386695	1.3117480	-0.5270511	1

E_{tot} = –925.15875713139 H

Coordinates of [Au(CF_3)F_2] on RI-B3LYP-D3/def2-TZVPP Level

xyz-Coordinates [Å] of the optimized minimum structure of [Au(CF_3)F_2].

7

Au	2.20755188580994	-1.19969376545462	0.00000000000000	79
F	0.41105050860525	-4.36929996693524	0.00000000000000	9
F	4.31337790822559	1.76345663419263	0.00000000000000	9
C	-1.21257449415516	0.67574610062594	0.00000000000000	6
F	-0.80137650969151	3.12502431304880	0.00000000000000	9
F	-2.45901464939702	0.00238334226124	-2.04713663284360	9
F	-2.45901464939702	0.00238334226124	2.04713663284360	9

E_{tot} = –673.04056490330 H

Coordinates of [Au(CF_3)$_3$] on RI-B3LYP-D3/def2-TZVPP Level

xyz-Coordinates [Å] of the optimized minimum structure of [Au(CF_3)$_3$].

13

Au	0.04719566441461	-1.12233322931971	0.03028927353388	79
C	-3.95033446217129	-1.37469420800382	-0.00872322407669	6
C	-0.06832374671078	2.81381072796679	-0.11518311269927	6
C	4.03074957445271	-1.46396478353996	0.10920220984482	6
F	-4.47788327211836	-3.84613964812156	0.55461107074308	9
F	-5.19385000458658	0.04463627360978	1.70600355067283	9
F	-5.02534037356166	-0.90704606747568	-2.27606697154130	9
F	-1.56053017339052	3.51262337321273	-1.99264397200624	9
F	-0.96976458490633	3.64844169890046	2.05916304044270	9
F	2.22491171582116	3.72355723121026	-0.48326464487953	9
F	5.23608929968403	-0.73325664413322	-2.01955388842153	9
F	5.20229233274008	-0.29621263509404	2.05304199557828	9
F	4.50478803033292	-3.99942208921198	0.38312467280897	9

E_{tot} = –1148.65383085260 H

Literature

[1] a) D. J. Adams, J. H. Clark, L. B. Hansen, V. C. Sanders, S. J. Tavener, *J. Fluorine Chem.* **1998**, *92*, 123; b) E. Schnell, E. G. Rochow, *J. Inorg. Nucl. Chem.* **1958**, *6*, 303; c) I. Ruppert, K. Schlich, W. Volbach, *Tetrahedron Lett.* **1984**, *25*, 2195; d) S. Martinez-Salvador, L. R. Falvello, A. Martin, B. Menjon, *Chem. Eur. J.* **2013**, *19*, 14540; e) M. A. Ellwanger, C. von Randow, S. Steinhauer, Y. Zhou, A. Wiesner, H. Beckers, T. Braun, S. Riedel, *Chem. Commun.* **2018**, *54*, 9301.
